# Insight into the Interactome of Intramitochondrial PKA Using Biotinylation-Proximity Labeling

**DOI:** 10.3390/ijms21218283

**Published:** 2020-11-05

**Authors:** Yasmine Ould Amer, Etienne Hebert-Chatelain

**Affiliations:** 1 Department of Biology, University of Moncton, Moncton, NB E1A 3E9, Canada; eyo9935@umoncton.ca; 2 Canada Research Chair in Mitochondrial Signaling and Physiopathology, University of Moncton, Moncton, NB E1A 3E9, Canada

**Keywords:** mitochondria, protein kinase A, BioID2, proteomics, serine/threonine phosphoprediction, TIM44

## Abstract

Mitochondria are fully integrated in cell signaling. Reversible phosphorylation is involved in adjusting mitochondrial physiology to the cellular needs. Protein kinase A (PKA) phosphorylates several substrates present at the external surface of mitochondria to maintain cellular homeostasis. However, few targets of PKA located inside the organelle are known. The aim of this work was to characterize the impact and the interactome of PKA located inside mitochondria. Our results show that the overexpression of intramitochondrial PKA decreases cellular respiration and increases superoxide levels. Using proximity-dependent biotinylation, followed by LC-MS/MS analysis and in silico phospho-site prediction, we identified 21 mitochondrial proteins potentially targeted by PKA. We confirmed the interaction of PKA with TIM44 using coimmunoprecipitation and observed that TIM44-S80 is a key residue for the interaction between the protein and the kinase. These findings provide insights into the interactome of intramitochondrial PKA and suggest new potential mechanisms in the regulation of mitochondrial functions.

## 1. Introduction

Mitochondria are crucial in the regulation of cell metabolism, proliferation and survival. These organelles are responsible for generating most of the cellular adenosine triphosphate (ATP) through oxidative phosphorylation (OXPHOS) which links oxidation of metabolic fuels to the production of ATP [1,2]. During this process, electrons are transferred from NADH or FADH_2_ to O_2_ by four electron carriers (enzymatic complexes named complex I to IV). Simultaneously, protons are pumped from the mitochondrial matrix to the intermembrane space establishing an electrochemical gradient across the inner mitochondrial membrane (IMM), which is ultimately used by the ATP synthase to generate ATP. Mitochondria are also involved in intracellular calcium homeostasis, generation of reactive oxygen species (ROS) and apoptosis [3].

Reversible phosphorylation is involved in the maintenance of mitochondrial functions and cellular homeostasis [4,5,6,7,8]. Several serine (S) and threonine (T) kinases translocate to mitochondria in particular conditions to phosphorylate mitochondrial substrates [9]. For instance, AMP-activated protein kinase (AMPK) and pyruvate dehydrogenase kinase (PDK) target cytochrome *c* and pyruvate dehydrogenase E1-α, respectively, to adapt mitochondrial functions during ischemia/reperfusion-induced injury [10,11,12].

Protein kinase A (PKA) is involved in numerous physiological processes, including metabolism, gene transcription, cell division, and cell differentiation [13]. Eucaryotic PKA holoenzymes are composed of two regulatory subunits (PKA-R) bound to two catalytic subunits (PKA-C). There are four PKA-R isoforms (i.e., RI-α, RI-β, RII-α and RII-β) and three PKA-C isoforms (i.e., C-α, C-β and C-γ). Binding of cAMP to the regulatory subunits induces dissociation of the holoenzyme and subsequent phosphorylation of key substrates by the free and active PKA-C [14]. Cyclic AMP is degraded by cAMP phosphodiesterases (PDEs). Phosphorylation of PDEs by PKA reduces cAMP levels and downregulates cAMP signaling in a negative feedback loop [15]. PKA is targeted to specific subcellular compartments by A-kinase anchoring proteins (AKAPs). PKA was one of the first kinase to be associated to phosphorylation of mitochondrial proteins [16], through the observation of cAMP-dependent phosphorylation both at the surface and inside mitochondria [17,18,19,20]. AKAPs such as AKAP-1, anchor PKA to the outer mitochondrial membrane (OMM) where it targets several proteins [21,22,23]. Notably, PKA phosphorylates the glutathione S-transferase alpha 4 protein on S189 to promote its interaction with the chaperone heat shock protein 70 (Hsp70), facilitate its translocation to mitochondria and increase its activity [24]. PKA also targets three subunits of the OMM import machinery, i.e., TOM22, TOM40 and TOM70, to blunt protein import into mitochondria [25,26,27]. Additionally, phosphorylation of BAD-S112/155, BAX-S60 and BIM-S83 by PKA inhibits apoptosis [28,29,30,31]. Phosphorylation of Drp1-S637 by PKA prevents mitochondrial fission [32,33].

Several intramitochondrial proteins were also shown to be phosphorylated by PKA. For instance, PKA phosphorylates COXIV-1-S58, preventing complex IV inhibition by ATP [34], whereas PKA-dependent phosphorylation of the ATPase inhibitory factor 1 (AIF1) on S39 impedes its binding to ATP synthase [35]. PKA also downregulates mtDNA replication and mitochondrial biogenesis by phosphorylating the mitochondrial transcription factor A, which impairs its ability to bind DNA and activate transcription [36]. Whether PKA targets these proteins in the cytosol before their translocation or directly within the organelle is, however, still debated. For instance, PKA can phosphorylate NDUFS4 in the cytosol to modulate its transport inside the organelle and the assembly of complex I [37]. Interestingly, the AKAP sphingosine kinase interacting protein (also named SKIP) tethers PKA in the intermembrane space and in the matrix where it interacts with MIC19 and MIC60 [38]. In addition, indirect evidence suggest that a soluble adenylyl cyclase is localized inside the mitochondrial matrix where it generates cAMP to directly activate intramitochondrial PKA and modulate ATP levels [39,40,41]. Therefore, it appears important to better describe the role of PKA localized inside mitochondria for which only few targets were identified so far.

The aim of this work was to examine the interactome of PKA localized in mitochondria. To address this, we generated a construct encoding PKA specifically targeted to the interface of the IMM and the matrix (mt-PKA) and examined its impact on mitochondrial activity. Our findings indicate that overexpressed mt-PKA alters mitochondrial functions and phosphorylates several mitochondrial proteins. Using proximity-dependent biotin identification, LC-MS/MS and in silico phospho-site prediction, we identified potential new substrates of mt-PKA including TIM44-S80. We also observed that TIM44-S80 phosphomutants impact OXPHOS and the physical interaction between TIM44 and mt-PKA.

## 2. Material and Methods

### 2.1. Cloning and Site-Directed Mutagenesis

PKA-Myc and mt-PKA-Myc constructs were generated as described [42]. Briefly, the mitochondrial leading sequence of COXVIIIa subunit was fused to the Myc-tagged PKA-C-α sequence [42]. The mt-PKA-BioID2-HA plasmid was generated by fusing the human influenza hemagglutinin (HA) tagged BioID2 sequence (Addgene plasmid #74224) to the mt-PKA-C-α sequence [43,44].

pcDNA3-TIM44-V5 was kindly provided by Pr. Elena Bonora (University of Bologna, Bologna, Italy). Site-directed mutagenesis was performed following the Q5^®^ High-Fidelity DNA Polymerase (ref. M0491, New England Biolabs, Ipswich, MA,) PCR protocol, using the following synthetic oligonucleotides, forward: 5′-AATGAAAGAAgcTATAAAAAAATTCCGTGACGAG-3′ with reverse 5′-TCTTTGTTTTTGGCTAATTC-3′, and forward 5′-AATGAAAGAAgaTATAAAAAAATTCCGTGACGAG-3′ with reverse 5′-TCTTTGTTTTTGGCTAATTC-3′ to generate the phosphomimetic (TIM44-S80D) and the phosphodeficient (TIM44-S80A) mutants of TIM44, respectively.

### 2.2. Cell Culture and Transient Transfection

HeLa and HEK cells were cultured in high glucose (4,5 g/L Dulbecco’s modified Eagle’s medium (DMEM) supplemented with 2 mM glutamine, 1 mM pyruvate, 10% (*v*/*v*) FBS and penicillin-streptomycin. Cells were kept at 37 °C in 5% CO₂ and 95% humidity. Cells were transiently transfected with polyethylenimine (PolySciences, Warrington, PA, USA) and analyzed 48 h following transfection.

### 2.3. Subcellular Fractionation

Isolation of subcellular fractions was performed as described previously [45]. Briefly, cells were harvested and resuspended in mitochondrial isolation buffer (250 mM sucrose, 1 mM EDTA, 5 mM HEPES, pH 7.4) supplemented with 1% protease inhibitor cocktail (Bioshop, ON, Canada), 2 mM sodium orthovanadate and 1 mM sodium fluoride. Cells were lysed with 15 strokes using a 25-gauge syringe on ice and centrifuged at 1500× *g* for 5 min (4 °C). The resulting supernatant (TCL) was centrifuged at 12,500× *g* for 10 min (4 °C). The obtained supernatant was considered as the cytosolic fraction (cyto), the pellet was resuspended in the mitochondrial buffer and a cycle of centrifugation at 1500× *g* and 12,500× *g* was repeated. The final pellet was considered as the mitochondria-enriched fraction (mito). Protein concentration was determined by Bradford assay [46].

### 2.4. Trypsin Sensitivity Assay

The trypsin sensitivity assay was carried out as described previously [47] with minor modifications. Briefly, isolated mitochondria were suspended in mitochondrial isolation buffer and incubated at 37 °C for 10 min in presence or absence of trypsin (0.5%) and triton X-100 (1%). Reaction was stopped by the addition of 1% of the protease inhibitor cocktail. Mitochondria were then centrifuged at 12,500× *g* at 4 °C for 10 min. The pellets were processed for SDS-PAGE.

### 2.5. Mitochondrial Subfractionation

Isolated mitochondria were resuspended in 1M HEPES-KOH buffer (pH 7.4) in the presence of 0.125, 0.25 or 0.5% of digitonin and incubated at room temperature with continuous shaking at 1000 rpm. After 15 min, samples were centrifuged at 12,500× *g* at 4 °C for 10 min. The resulting pellets and supernatants were processed for SDS-PAGE. The presence of proteins from the different mitochondrial subcompartments in pellets and supernatants was analyzed using immunoblotting.

### 2.6. SDS-PAGE and BN-PAGE

Samples were analyzed by SDS-PAGE at 200 V using 7, 10 or 12% SDS-polyacrylamide mini-gel containing 0.35% (*v*/*v*) of 2,2,2-trichloroethanol for staining loaded proteins. For BN-PAGE, cells were solubilized in 2% digitonin for 10 min on ice and centrifuged during 10 min at 12,500× *g*. Supernatant was then supplemented with 0.5% Coomassie G-250 and samples were loaded in 4–16% Bis-Tris gels (LifeTechnologies, Pleasanton, CA, USA). Voltage was initially set to 150 V for 45 min and at 300 V during the following 30 min. Proteins were then transferred to polyvinylidene difluoride (PVDF) membranes. The total amount of proteins loaded in SDS-PAGE gels was visualized and quantified using the stain-free method, as described [48]. Briefly, 2,2,2-trichloroethanol interacts with tryptophan in loaded proteins and induce UV light-induced fluorescence which can be visualized on a 300 nm transilluminator. Immunolabeling can then be normalized to the total UV light-induced fluorescence (corresponding to the total protein load) instead of using unique proteins (such as tubulin or actin) as loading control. Membranes were blocked for 1 h in TBS-T (50 mM Tris-Cl, pH 7.6; 150 mM NaCl, 0.1% Tween) containing 5% BSA or 5% skim milk and incubated with primary antibodies overnight at 4 °C. Protein immunodetection was performed using primary antibodies directed against PKA C-α (Cell Signaling; 4782S), PKA RI-α/β (Cell Signaling; 3927), Phospho-PKA Substrate (Cell Signaling; 9624S), α-Tubulin (Cell Signaling; 2144) SDHa (Abcam; ab14715), SOD2 (Santa Cruz; sc-30080), NDUF9a (Abcam; ab14713), Cytochrome c (Abcam; ab110325), TOM20 (Santa Cruz; (F-10) sc-17764), UQCRC2 (Abcam; ab14745), PAM16 (Abcam; Ab184157), Hsp60 (Santa Cruz; sc-13115), Hsp70 (Santa Cruz; sc-24), V5 (Cell Signaling; 13202S), Myc (Cell Signaling; 2276S), ATP5a (Abcam; ab14730), MFN2 (Cell signaling; 9482S), ERp57 (R&Dsystems; AF8219). Membranes were then incubated for 1 h with peroxidase-conjugated antimouse or antirabbit secondary antibodies. Immunoblots were visualized by chemiluminescence using the ChemiDoc Touch imaging system (Biorad, Irvine, CA, USA).

### 2.7. Confocal Microscopy Imaging

Cells seeded on 18-mm round glass coverslips, transfected as indicated were placed on the stage of the Olympus FV3000 confocal fluorescence microscope (Tokyo, Japan) and imaged using a 60X oil objective (UPLAN 60× oil, 1.35 NA, Olympus), and appropriate excitation laser and filters. For each experiment, 25 cells were randomly selected and analyzed. Stacks of 30 images separated by 0.2 μm along the Z axis were acquired. Three-dimensional reconstruction and volume rendering of the stacks were carried out with the appropriate plug-in of ImageJ (NIH, Bethesda, MD, USA).

## 3. Measurement of the Mitochondrial Membrane Potential, Mitochondrial Mass and ROS Levels

Mitochondrial membrane potential was examined using tetramethylrhodamin, methyl ester (TMRM, LifeTechnologies, Pleasanton, CA, USA). Briefly, cells expressing the different constructs were rinsed with PBS and incubated with 100 nM TMRM during 15 min at 37 °C. Cells coincubated with FCCP (6 µM during 15 min) had no TMRM fluorescence (data not shown).

Oxidative stress was evaluated using MitoSox™. Briefly, cells expressing the different constructs were incubated with 5 µM MitoSox™ during 45 min at 37 °C in 5% CO₂ and 95% humidity. Pretreatment of cells with 0.5 µM rotenone and 2.5 µM antimycin A during 15 min significantly increased MitoSox™ labeling (data not shown).

Mitochondrial mass was evaluated using Mitotracker Green™. Cells were incubated with 200 nM Mitotracker Green™ for 30 min at 37 °C.

TMRM, MitoSox™ and Mitotracker Green™ fluorescence were examined using the EVOS FL Auto 2 imaging system with a 40× objective (LPLAN 40×, 0.65NA, EVOS). For each independent experiment, fluorescence intensity was quantified in 25 cells using ImageJ (NIH, MD, USA).

## 4. Measurement of Oxygen Consumption

Oxygen consumption assays were performed using the high-resolution respirometry system Oroboros™ Oxygraph-2k (Innsbruck, Austria). Cell respiration was measured with 5 × 10^5^ cells mL^−1^ at 37 °C in 2 mL chambers at a stirring rate of 750 rpm. Three different states of endogenous respiration with intact cells were measured: (i) basal respiration representing the endogenous physiological coupled state, (ii) respiration with oligomycin (2 μg.mL^−1^) representing the non-coupled resting respiration, and (iii) maximal uncoupled respiration induced by FCCP (0.5 μM steps with 2.5 μM final concentration) providing the maximal respiration.

## 5. Proximity-Dependent Biotinylation and Streptavidin Bead Pulldown Assay

4 × 10^6^ HEK cells expressing pcDNA or mtPKA-BioID2-HA were incubated with 0 or 50 µM biotin during 24 h. Cells were then rinsed twice with PBS and lysed in 500 µL of lysis buffer (50 mM Tris, pH 7.4, 500 mM NaCl, 0.4% SDS and 1 mM dithiothreitol) supplemented with 1% protease inhibitor cocktail, 2 mM sodium orthovanadate and 1 mM sodium fluoride. Lysed cells were sonicated at 60% amplitude three times for 2 s. An equal volume of ice-cold 50 mM Tris, pH 7.4 was then added before centrifugation at 15,000× *g* for 15 min. 20 µL of the supernatant was processed for SDS-PAGE to verify the efficiency of protein biotinylation. The remaining supernatant was incubated with 25 µL of streptavidin beads at 4 °C overnight under constant agitation. Beads were then washed twice with lysis buffer and centrifuged at 1500× *g* for 5 min. Supernatant was then removed and beads were resuspended in 100 µL of 50 µM ammonium bicarbonate. 20 µL of beads were processed for immunoblotting to verify the efficiency of protein biotinylation. Proteins on beads were rinsed three times with 50 mM ammonium bicarbonate buffer and kept at −80 °C prior to mass spectrometry analyses.

## 6. LC-MS/MS Analysis

### 6.1. Tryptic Digestion

Mass spectrometry was performed at the Proteomics platform of the CHU de Quebec Research Center (Quebec City, QC, Canada). Proteins on beads were suspended in 25 µL of 50 mM ammonium bicarbonate containing 1 µg trypsin and incubated overnight at 37 °C. Trypsin reaction was stopped by acidification with 3% acetonitrile, 1% trifluoroacetic acid, 0.5% acetic acid. Beads were then removed by centrifugation. Peptides were purified from supernatant on stage tip (C18) and vacuum-dried before MS injection. Samples were solubilized into 5 µL of 0.1% formic acid.

### 6.2. Peptide Separation and MS Detection

Peptide samples were separated by online reversed-phase (RP) nanoscale capillary liquid chromatography (nanoLC) and analyzed by electrospray mass spectrometry (ES MS/MS). The experiments were performed with an Ekspert NanoLC425 coupled to a 5600+ mass spectrometer (Sciex, Framingham, MA, USA) equipped with a nanoelectrospray ion source. Peptide separation took place on a self-packed picofrit column with reprosil 3u, 120A C18, 17 cm × 0.075 mm internal diameter, (Dr Maisch HPLC GmbH, Ammerbuch, Germany). Peptides were eluted with a linear gradient from 5–35% solvent B (acetonitrile, 0.1% formic acid) in 35 min, at 300 nL/min. Mass spectra were acquired using a data dependent acquisition mode using Analyst software version 1.7. Each full scan mass spectrum (400 to 1250 m/z) was followed by collision-induced dissociation of the twenty most intense ions. Dynamic exclusion was set for a period of 12 s and a tolerance of 100 ppm.

### 6.3. Database Searching

MGF peak list files were created using Protein Pilot version 4.5 software (Sciex, Framingham, MA, USA). MGF sample files were then analyzed using Mascot (Matrix Science, London, UK; version 2.5.1).

### 6.4. Criteria for Protein Identification

Scaffold (version Scaffold_4.7.1, Proteome Software Inc., Portland, OR, USA) was used to validate MS/MS based peptide and protein identification.

### 6.5. Coimmunoprecipitation

Whole cell extracts from HEK293 cells transiently transfected with the indicated plasmids were resuspended in lysis buffer (PathScan^®^ Sandwich ELISA 1X, Cell Signaling #7018) supplemented with 1% protease inhibitor cocktail, 2 mM sodium orthovanadate and 1 mM sodium fluoride. Samples were then centrifuged at 12,500× *g* during 10 min (4 °C). Immunoprecipitation was performed by incubating 2 mg protein with anti-V5 tag antibody (Cell Signaling, D3H8Q Rabbit mAb #13202) under overnight agitation at 4 °C. Then, 20 µL of protein A/G plus-agarose (Santa Cruz, sc-2003) beads were added and incubated for 4 h at 4 °C. Beads were washed three times with lysis buffer and elution was performed with SDS-PAGE sample buffer for 5 min at 95 °C. Samples were then processed for Western blotting.

### 6.6. Cycloheximide Chase Assay

HeLa cells expressing different constructs were treated with cycloheximide (40 µg/mL) during 0, 10, 20, 30, 60 and 180 min. Cells were then harvested and processed for immunoblotting to analyze the stability of TIM44-V5 protein variants.

### 6.7. Statistical Analyses

Data are presented as mean ± s.e.m. Statistical analyses were performed using GraphPad Prism v. 8.0 (GraphPad, San Diego, CA, USA). Data were analyzed using one-way or two-way ANOVA, as appropriate. *p* < 0.05 was considered statistically different.

## 7. Results

### 7.1. Functional Impact of mt-PKA on Mitochondria

**Figure 1:**
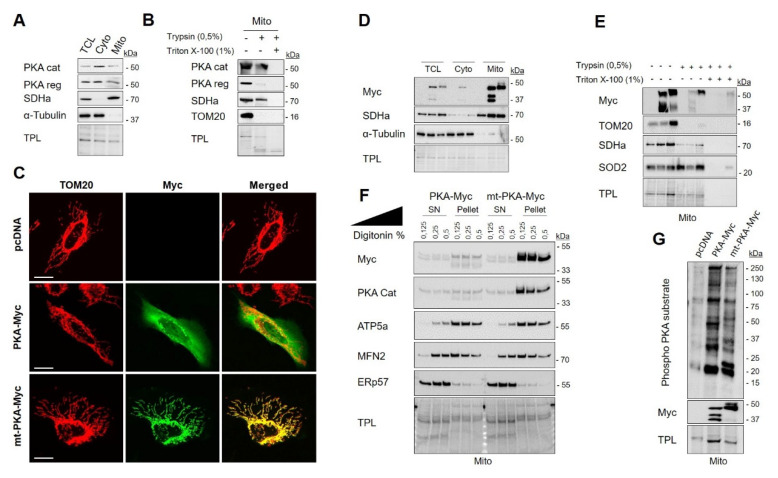
Protein kinase A (PKA) catalytic subunit α is localized within mitochondria of HeLa cells. (**A**) Representative immunoblots (*n* = 3) of PKA catalytic (cat) subunit, PKA regulatory (reg) subunit, the mitochondrial protein SDHa and of the cytosolic protein α-Tubulin with corresponding total protein load (TPL) in total cell lysate (TCL), cytosolic (Cyto) and mitochondrial (Mito) fractions isolated from HeLa cells. (**B**) Representative immunoblots (*n* = 3) of PKA cat, PKA reg, SDHa and TOM20 with corresponding total protein load (TPL) in mitochondrial fractions isolated from HeLa cells and treated as indicated. (**C**) Representative micrographs (*n* = 6) of HeLa cells expressing empty vector (pcDNA), PKA-Myc or mt-PKA-Myc and labelled with anti-Myc (green) and anti-TOM20 (red) as a mitochondrial marker. Scale bars = 5 μm. (**D**) Representative immunoblots (*n* = 3) of Myc, SDHa and α-Tubulin with corresponding total protein load (TPL) in TCL, cyto and mito fractions isolated from HeLa cells expressing empty vector (pcDNA), PKA-Myc or mt-PKA-Myc. (**E**) Representative immunoblots (*n* = 3) of Myc, TOM20, SDHa and SOD2 with corresponding total protein load (TPL) in mitochondrial enriched fractions isolated from HeLa cells expressing empty vector (pcDNA), PKA-Myc or mt-PKA-Myc and treated as indicated. (**F**) Representative immunoblots (*n* = 3) of Myc, PKA Cat, ATP5a, MFN2 and ERp57 with corresponding TPL in pellet and supernatant (SN) obtained after treatment of mitochondria isolated from HeLa cells expressing PKA-Myc or mt-PKA-Myc and treated as indicated. (**G**) Representative immunoblots (*n* = 5) of phospho-PKA substrates and Myc with corresponding total protein load (TPL) in mitochondrial enriched fractions isolated from HeLa cells expressing empty vector (pcDNA), PKA-Myc or mt-PKA-Myc.

PKA influences mitochondrial functions through phosphorylation of several targets [49,50,51]. PKA signaling has distinct targets and biological effects inside and outside mitochondria [52]. The role of PKA localized inside mitochondria remains, however, poorly understood. We first examined endogenous expression and subcellular localization of PKA in HeLa cells. Previous works have shown that PKA subunits are present in multiple cellular compartments, including mitochondria [16,53,54]. Similarly, our findings showed that PKA-RI-α/β and PKA-C-α subunits are present in both cytosolic and mitochondria-enriched fractions (Figure 1A). To examine the localization of PKA subunits among the mitochondrial subcompartments, we then performed trypsin sensitivity on mitochondria-enriched fractions. Treatment of mitochondria with trypsin led to strong degradation of the RI-α/β subunits similar to the OMM protein TOM20 (Figure 1B). In contrast, the PKA-C-α subunits resisted to trypsin proteolysis similar to the IMM/matrix protein SDHa (Figure 1B). These results thus suggest that a significant pool of PKA-C-α resides inside mitochondria of HeLa cells.

In order to decipher the specific role of PKA catalytic subunit α localized inside mitochondria, we generated two constructs: (i) Myc tagged PKA-C-α (named hereafter PKA-Myc) and (ii) Myc tagged PKA-C-α fused to the mitochondrial leading sequence of COXVIIIa, a protein localized at the interface of the IMM and the matrix [55] (named hereafter mt-PKA-Myc). Immunodetection of Myc and TOM20 by confocal microscopy showed that PKA-Myc spreads throughout the cell whereas mt-PKA-Myc is specifically targeted to mitochondria (Figure 1C). Subcellular fractionation confirmed that both constructs express PKA in specific subcellular compartments (Figure 1D). However, the resolution of conventional confocal microscopes is not sufficient to analyze the submitochondrial distribution of proteins [56]. We thus performed trypsin sensitivity assays on mitochondria-enriched fractions of HeLa cells expressing the different constructs. Similar to TOM20, PKA-Myc was mostly degraded by trypsin, whereas mt-PKA-Myc was only partly degraded by trypsin, similar to SDHa and SOD2 (Figure 1E). Similar findings were obtained when mitochondria-enriched fractions were solubilized by digitonin. As shown in Figure 1F, the OMM protein MFN2 and the IMM protein ATP5a were dose-dependently released in the supernatant after treatment with digitonin. Immunoblotting of Myc in the same conditions further suggest that mt-PKA-Myc is mostly recruited at the mitochondrial matrix and/or IMM (Figure 1F). Considering that mitochondria-enriched fractions are often contaminated by the endoplasmic reticulum (ER), we also examined the release of the ER protein ERp57 after treatment with digitonin. The obtained findings further suggest that mt-PKA is not localized in ER (Figure 1F). Using immunoblotting, we confirmed that expression of both PKA constructs increased phosphorylation of serine and threonine on RRXS/T motifs of mitochondrial proteins in HeLa cells (Figure 1G). Overall, these findings indicate that the mt-PKA-Myc construct is an appropriate tool to characterize the role of PKA-C-α localized at the interface of matrix and IMM.

**Figure 2:**
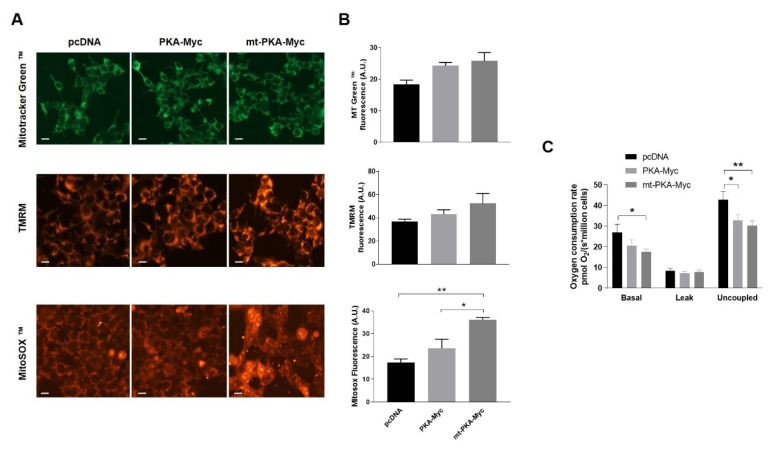
Overexpression of PKA alters mitochondrial activity. (**A**) Representative micrographs of HeLa cells expressing empty vector (pcDNA), PKA-Myc or mt-PKA-Myc and labeled with Mitotracker Green™ (MT Green), TMRM and MitoSOX™. Scale bars = 10 μm. (**B**) Quantification of Mitotracker Green™, TMRM and MitoSOX™ fluorescence intensity (*n* = 3, with 25 random cells per experiment). A.U.: Arbitrary Units. (**C**) Oxygen consumption rates of HeLa cells expressing empty vector (pcDNA), PKA-Myc or mt-PKA-Myc (*n* = 3). Data are presented as mean ± s.e.m. analyzed by one-way ANOVA followed by Tukey’s test (* *p* < 0.05), (** *p* < 0.01).

In order to evaluate the functional impact of mt-PKA, HeLa cells were transfected with pcDNA, PKA-Myc or mt-PKA-Myc and labeled with different probes to examine mitochondrial physiology. Live imaging of cells stained with MitoTracker Green™ and TMRM indicated no difference of mitochondrial mass and membrane potential among cells expressing pcDNA, PKA-Myc or mt-PKA-Myc (Figure 2A,B). MitoSOX™ fluorescence indicated higher levels of superoxide in cells expressing mt-PKA-Myc (Figure 2A,B). Overexpression of mt-PKA-Myc significantly decreased basal and uncoupled respiration rates (Figure 2C). PKA-Myc had a similar impact on respiration although only uncoupled respiration was significantly reduced by this construct (Figure 2C). Overall, these findings suggest that the overexpression of intramitochondrial PKA-C-α alters mitochondrial functions.

### 7.2. Identification of Potential Substrates of mt-PKA

**Figure 3:**
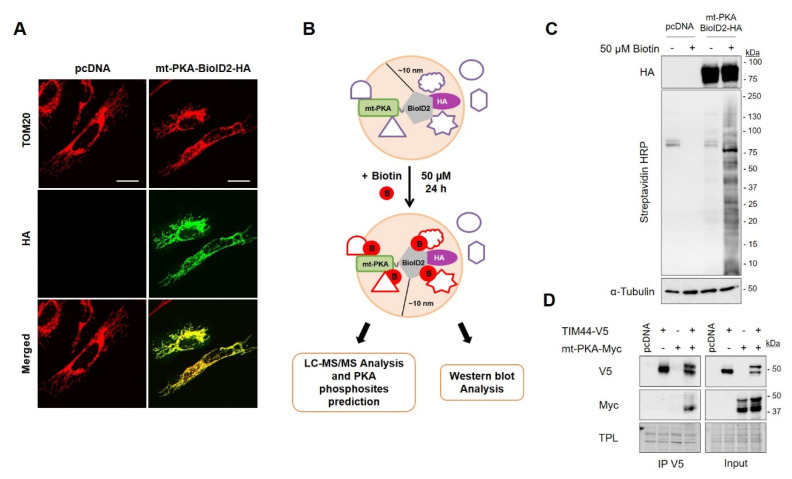
Biotinylation-based proximity labeling of potential interactors of mt-PKA. (**A**) Representative (*n* = 5) micrographs of HeLa cells expressing empty vector (pcDNA) or mt-PKA-BioID2-HA and labeled with HA (green) and TOM20 (red) as a mitochondrial marker. Scale bars = 10 μm. (**B**) Schematic representation of the biotinylation-based proximity labeling combined with mass spectrometry analysis showing that mt-PKA-BioID2-HA add biotin to proteins located within a radius of 10 nm. (**C**) Representative immunoblots (*n* = 5) of HA, Streptavidin coupled to horseradish peroxidase (HRP) and α-Tubulin in total cell lysates of HEK cells expressing empty vector (pcDNA) or mt-PKA-BioID2-HA and treated as indicated. (**D**) Representative immunoblots (*n* = 4) of V5 and Myc after immunoprecipitation (IP) of V5 from HEK cells expressing pcDNA, TIM44-V5 and/or mt-PKA-Myc. (TPL: total protein load).

In order to identify the proteins involved in the regulation of mitochondrial functions by mt-PKA-Myc, we used proximity-dependent biotin identification (BioID) [44]. We first fused mt-PKA to HA tagged promiscuous biotin ligase BioID2. Immunofluorescence confirmed the mitochondrial localization of mt-PKA-BioID2-HA since HA colocalized with the mitochondrial protein TOM20 (Figure 3A). Addition of biotin (50 µM) to the culture medium of cells expressing mt-PKA-BioID2-HA promotes biotinylation of proteins within a ~10 nm radius of the BioID2 (Figure 3B) [43]. Immunoblotting confirmed that biotin treatment triggered protein biotinylation in cells expressing mt-PKA-BioID2-HA (Figure 3C). Biotinylated proteins were then pulled down using streptavidin-coated beads. Biotinylated-purified proteins from cell expressing pcDNA or mt-PKA-BioID2-HA and treated with biotin were submitted to on-bead tryptic digestion and LC/MS-MS analysis. A total of 1898 proteins were first identified including 190 mitochondrial proteins (Table S1). Filters were then applied to increase robustness of MS data and optimize the identification of mitochondrial proteins most likely interacting with PKA. We first selected the 537 proteins for which a minimum of five peptides was detected by LC/MS-MS in at least one sample, using protein and peptide FDR thresholds of 95% (Table S2). To minimize the selection and identification of proteins biotinylated independently of mt-PKA-BioID2-HA, we then selected proteins with a minimum difference of five peptides between cells expressing mt-PKA-BioID2-HA and pcDNA. We thus selected 208 proteins following this criterion (Table S3), including 33 mitochondrial proteins (Table S4). It is possible that the mt-PKA-BioID2-HA biotinylated proteins within a radius of 10 nm not directly interacting with mt-PKA. We thus performed in silico analysis to identify proteins with phospho-PKA binding motifs and potentially phosphorylated by mt-PKA. PKA phosphorylation of NDUFS4-S173 was used as a positive control (Table S5) [37].

Using the phospho-site prediction software pkaPS (http://mendel.imp.univie.ac.at/sat/pkaPS) [57], NetPhos 3.1 (http://www.cbs.dtu.dk/services/NetPhos/) [58] and GPS 3.0 (http://gps.biocuckoo.org/online.php) [59], we identified 21 mitochondrial proteins for which at least one phospho-site was predicted simultaneously by the three software (Table 1 and Table S5).

Overall, our approach identified 21 mitochondrial proteins potentially targeted by mt-PKA, involved in various molecular functions ranging from metabolite exchange, protein synthesis and degradation, to tricarboxylic (TCA) cycle, mitochondrial dynamics, protein import and cell survival (Table 1).

### 7.3. PKA Interacts with TIM44

In silico analyses suggest that PKA could phosphorylate TIM44-S80 (Table 1). To confirm the interaction of TIM44 with mt-PKA, we generated V5 tagged TIM44 constructs. Interestingly, immunoprecipitation of V5 from whole cell lysates of HEK cells expressing TIM44-WT-V5 and mt-PKA-Myc showed that mt-PKA-Myc coimmunoprecipitated with TIM44-V5 (Figure 3D), confirming the physical interaction between the two proteins.

### 7.4. Impact of TIM44-S80 Phosphomutants on Mitochondria

**Figure 4:**
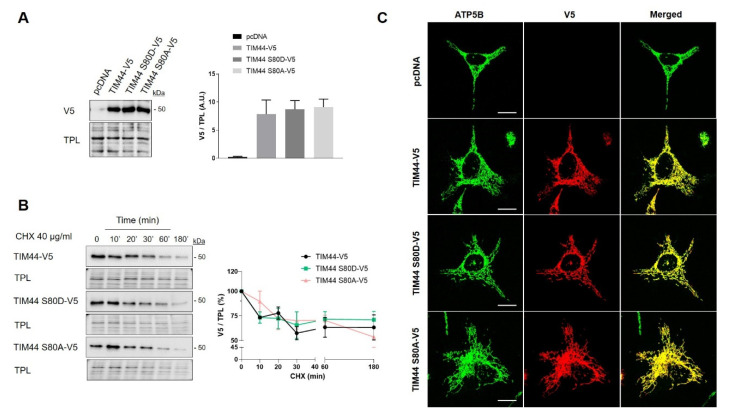
Impact of TIM44-S80 phosphomutations on the stability and localization of TIM44-V5. (**A**) Left, representative immunoblots (*n* = 5) of V5 in total cell lysates of HeLa cells expressing empty vector (pcDNA), TIM44-V5, TIM44 S80D-V5 or TIM44 S80A-V5. Right, quantification of V5 levels normalized by total protein load (TPL) shown on left (A.U.: arbitrary unit). (**B**) Left, representative immunoblots (*n* = 5) and right, corresponding quantification of normalized V5 levels to total protein load (TPL) in total cell lysates of HeLa cells expressing empty vector (pcDNA), TIM44-V5, TIM44 S80D-V5 or TIM44 S80A-V5 and treated with cycloheximide (CHX) as indicated. (**C**) Representative micrographs (*n* = 5) of HeLa cells expressing empty vector (pcDNA), TIM44-V5, TIM44 S80D-V5 or TIM44 S80A-V5 and labeled with V5 (red) and ATP5B (green) as a mitochondrial marker. Scale bars = 10 μm.

The role of TIM44-S80 phosphorylation in mitochondrial physiology was then examined using phosphomimetic TIM44-S80D-V5 and phosphodeficient TIM44-S80A-V5 mutants. Immunoblotting revealed similar levels of TIM44-V5 variants, suggesting that TIM44-S80 phosphomutations do not impact on TIM44 level (Figure 4A). Cycloheximide treatment followed by Western blotting showed similar degradation rates of TIM44-V5 and phosphomutants (Figure 4B), indicating that the stability of TIM44-V5 is not affected by TIM44-S80 phosphomutations. Similarly, TIM44-S80 variants were all correctly imported inside mitochondria since they colocalized with the mitochondrial protein ATP5B (Figure 4C). Altogether, these findings suggest that the potential phosphorylation of TIM44-S80 would not alter TIM44 homeostasis.

**Figure 5:**
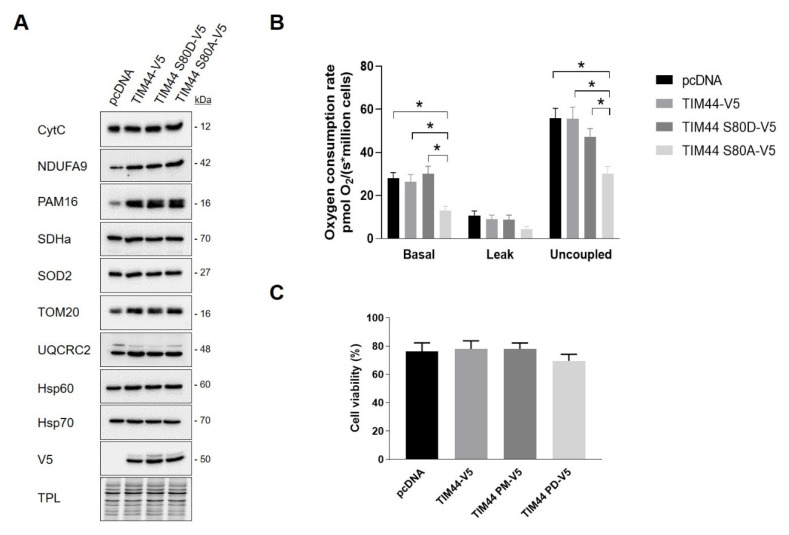
Impact of TIM44-S80 phosphomutants on mitochondrial content, cellular respiration and viability. (**A**) Representative immunoblots (*n* = 4) of mitochondrial proteins CytC, NDUFA9, PAM16, SDHa, SOD2, TOM20, UQCRC2, Hsp60 and Hsp70 in total cell lysates of HeLa cells expressing empty vector (pcDNA), TIM44-V5, TIM44 S80D-V5 or TIM44 S80A-V5. See Figure S1A for quantifications. (**B**) Basal, leak and uncoupled cellular respiration (*n* = 6) of HeLa cells expressing empty vector (pcDNA), TIM44-V5, TIM44 S80D-V5 or TIM44 S80A-V5. Data were analyzed by two-way ANOVA followed by Tukey’s post hoc test (* *p* < 0.05). (**C**) Cell viability (*n* = 6) determined by trypan blue dye exclusion assay of HeLa cells expressing empty vector (pcDNA), TIM44-V5, TIM44 S80D-V5 or TIM44 S80A-V5.

In order to assess the impact of TIM44-S80 phosphomutations on mitochondria, several proteins located in different mitochondrial compartments and involved in different mitochondrial functions were examined in HeLa cells expressing pcDNA or TIM44 variants. Immunoblottings showed that the level of these proteins remained unchanged among TIM44 mutants, suggesting that the phosphomutations of TIM44-S80 do not impact on mitochondrial content (Figure 5A and Figure S1A). Next, we examined the impact of these phosphomutations on cellular respiration and viability. We observed that the expression of the phosphodeficient TIM44-S80A-V5 mutant significantly decreased basal and uncoupled respiration rates (Figure 5B). These results suggest that phosphorylation of TIM44-S80 is needed to maintain optimal respiration. Trypan blue exclusion assay revealed that phosphomutants of TIM44-S80 did not affect cellular viability (Figure 5C). Overall, our findings suggest that the phosphorylation status of TIM44-S80 could impact on OXPHOS.

**Figure 6:**
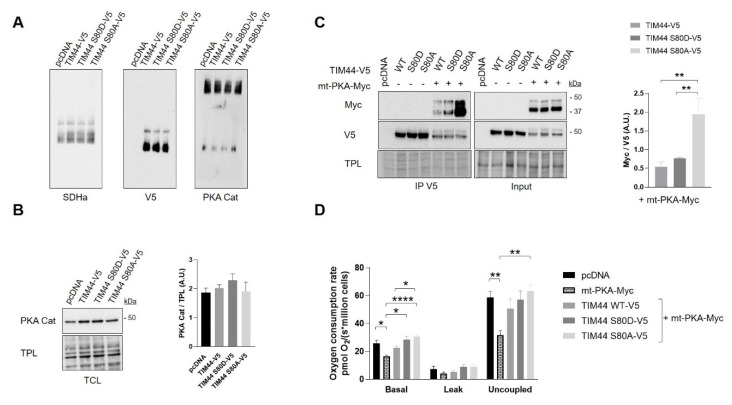
Functional interaction between TIM44-V5 phosphomutants and mt-PKA. (**A**) Representative BN-PAGE immunoblots (*n* = 4) of SDHa, V5 and PKA catalytic (Cat) subunit in TCLs of HeLa cells expressing empty vector (pcDNA), TIM44-V5, TIM44 S80D-V5 or TIM44 S80A-V5. See Figure S1B for quantifications. (**B**) Left, representative immunoblots (*n* = 4) of PKA catalytic (Cat) subunit levels and (right) corresponding quantifications on total protein loads (TPL) in total cell lysates of HeLa cells expressing empty vector (pcDNA), TIM44-V5, TIM44 S80D-V5 or TIM44 S80A-V5. (**C**) Left, representative immunoblots (*n* = 5) and (right) quantification of coimmunoprecipitated V5 with Myc from total cell lysates of HEK cells expressing pcDNA, TIM44 phosphomutants and/or mt-PKA-Myc. TPL: total protein load. Data were analyzed by one-way ANOVA followed Tukey’s post hoc test (** *p* < 0.01). (**D**) Basal, leak and uncoupled cellular respiration (*n* = 5) of HeLa cells expressing empty vector (pcDNA), mt-PKA-Myc, or co-expressing TIM44-V5, TIM44 S80D-V5 and TIM44 S80A-V5 with mt-PKA-Myc. Data were analyzed by two-way ANOVA test followed by Tukey’s post hoc test (* *p* < 0.05, ** *p* < 0.01, **** *p* < 0.0001).

We then examined the impact of TIM44-S80 phosphomutations on the functional relationship linking TIM44 to PKA. Immunoblottings from BN-PAGE first revealed similar levels of native complexes containing TIM44-V5 and the catalytic subunit α of PKA (Figure 6A and Figure S1B). Similarly, immunoblottings from SDS-PAGE showed no change in levels of the catalytic subunit α of PKA (Figure 6B) and of PKA activity (Figure S1C) among cells expressing the different TIM44-V5 constructs. However, TIM44-V5 levels globally decreased in presence of mt-PKA-Myc (Figure 6C and Figure S1D). Mt-PKA-Myc coimmunoprecipitated at higher levels with the phosphodeficient TIM44-S80A-V5 (Figure 6C). These results demonstrate that serine to alanine substitution at position 80 strengthens the interaction of TIM44-V5 to mt-PKA-Myc, suggesting that TIM44-S80 is involved in the physical interaction between the protein and PKA.

Interestingly, we observed that expression of TIM44-WT only partly and non-significantly rescued the mtPKA-dependent decrease of cellular respiration (Figure 6D). Also, both phosphomutants rescued, at least partly, the decrease of basal and uncoupled respiration induced by the mt-PKA overexpression (Figure 6D) without any effect on cell viability (Figure S1E). These findings suggest that mt-PKA could affect respiration via TIM44. It is however difficult to link these effects to the potential phosphorylation of TIM44-S80 considering that both phosphomutants reversed the alteration of respiration induced by the overexpression of mt-PKA.

## 8. Discussion

Although it is still debated, evidence suggest that PKA is localized within mitochondrial matrix where it can directly target proteins and modulate the activity of the organelle [38,39]. The aim of the present work was to characterize the interactome of PKA specifically targeted to the mitochondrial matrix using BioID. Our findings indicate that the overexpression of mt-PKA decreases cellular respiration and increases superoxide levels. Twenty-one mitochondrial proteins were then identified as potential substrates of mt-PKA using BioID, LC-MS/MS and in silico phospho-site prediction. Using coimmunoprecipitation, we confirmed the interaction of mt-PKA-Myc with TIM44-V5. The phosphodeficient mutant of TIM44-S80 negatively impacted cellular respiration and strengthened the interaction between mt-PKA and TIM44 constructs. Future studies will be needed to understand whether potential phosphorylation of TIM44-S80 affects the functions of the protein and its capacity to import proteins across the IMM.

PKA-C-α and PKA RI-α/β subunits were detected in mitochondria-enriched fractions of HeLa cells, although only the PKA-C-α subunit seems localized inside the organelle. These results are consistent with previous findings demonstrating the localization of AKAP121, PKA-RII and PKA-C at the IMM/matrix in both isolated mitochondria and cardiomyocytes [16]. Thus, we can speculate that the PKA-C-α subunits are coupled with PKA-RII and not PKA-RI subunits in mitochondria of Hela cells. Future studies should provide insights into the specific composition and distribution of PKA holoenzymes among the mitochondrial subcompartments of different cell types.

The present work shows that the overexpression of mt-PKA decreased OXPHOS and increased mitochondrial ROS levels (Figure 2A,B). Several studies previously showed that PKA impacts on mitochondrial activity via direct phosphorylation of OXPHOS components. Notably, PKA regulates OXPHOS via phosphorylation of NDUFS4, which is important to allow import of this protein inside the organelle and assembly of complex I [37]. PKA-mediated phosphorylation of NDUFA1-S55 is also necessary for appropriate complex I assembly [60,61]. In the presence of a high ATP/ADP ratio, PKA phosphorylates subunits of complex IV subunits and promotes its inhibition by ATP, consequently allowing a decrease in membrane potential and an increase in ROS production [34,62]. Mitochondrial PKA modulates complex V activity through phosphorylation of its inhibitor AIF1 [35]. These findings illustrate that PKA can modulate metabolism via direct phosphorylation of multiple OXPHOS components, although it is not clear whether these proteins are phosphorylated directly inside the organelle. The present work did not however identify proteins involved in OXPHOS as potential PKA substrates, suggesting that PKA can also modulate mitochondrial activity upstream of OXPHOS. For instance, GDH1 and ACL which are involved in the regulation of TCA cycle were targeted by mt-BioID2-HA. Deletion of the yeast PKA catalytic subunit Tpk3p reduces respiratory activity [63], whereas increased Tpk3p activity is sufficient to induce formation of dysfunctional mitochondria with striking morphological abnormalities that produce high levels of ROS [49]. These mitochondrial dysfunctions are induced by transcriptional changes that inhibit mitochondrial biogenesis, alter the electron transport system and inhibit stress response mechanisms [49]. Interestingly, excess ROS induce the sequestration of the PKA-C-α subunit into the matrix, leads to the hyper-phosphorylation of complex IV subunits I, IVi1, and Vb and reduces the activity of complex IV under hypoxia/ischemia [64,65]. Since these proteins were not identified here, the alterations of ROS and cellular respiration induced by the overexpression of mt-PKA observed in the present work were likely linked to different mechanisms. Overall, our findings suggest that the overexpression of mt-PKA impacted metabolism not via phosphorylation of OXPHOS components.

Using BioID, LC-MS/MS and in silico phospho-site prediction, we identified 21 potential substrates of mt-PKA. These proteins are involved in various processes such as import machinery, stress response, mitochondrial dynamics, TCA cycle, protein synthesis and degradation as well cell survival (Table 1). Several identified potential targets are involved in mitochondrial protein turnover (such as CLPX, IleRS, TRMT10C, LRP130, RNS4I, ATAD3A) and stress response (such as Hsp60, TRAP1, TCP-1-eta, P5CR1, SHMT2). For instance, P5CR1 has been reported to antagonize oxidative insults and promote cell survival [66]. SHMT2 is required for the assembly of complex I and the maintenance of mitochondrial respiration [67]. Expression of SHMT2 is altered by OXPHOS dysfunction [68] and is involved in protective response to mitochondrial toxicity [69]. Proteins involved in the mitochondrial unfolded stress response (UPR^mt^) were also identified as potential targets of mt-PKA, including the chaperones Hsp60 and TRAP1 [70] as well as the protease CLPX [71]. This stress response signals from perturbed mitochondria to increase the expression of genes encoding mitochondrial proteins involved in quality control [72,73]. UPR^mt^ protects cells from diverse mitochondrial stresses, including OXPHOS dysfunction and mitochondrial protein misfolding [74]. Overall, the potential substrates of mt-PKA identified here are involved in multiple steps of the mitochondrial proteins stress-responses and could be linked to the alterations of mitochondrial metabolism induced by the overexpression of mt-PKA.

Two-third of the potential targets of mt-PKA identified in this study (AKAP1, TOM70, TIM44, TRAP1, CLPX, TRMT10C, LRP130, SHMT2, ACL, ATAD3A, MIC19, HAX-1, MAVS) overlap with interactors of the mitochondrial prohibitin (PHB) complex. PHBs are IMM proteins that form ringlike structures composed of multiple PHB1 and PHB2 subunits. The PHB complex functions as a membrane scaffold important for the recruitment and stability of proteins within mitochondria [75]. For instance, PHBs control OPA-1 processing to regulate mitochondrial fusion [76]. They have also been involved in the organization and stability of mitochondrial nucleoids via their interaction with TFAM, mtSSB [77,78], and mtDNA-binding proteins such as ATAD3 [79]. Interestingly, deficiency in PHBs alters the assembly of (super)complexes of the electron transport system [80,81,82] and increases ROS production [83,84], suggesting that the metabolic alterations induced by the overexpression of mt-PKA observed here could be linked to PHB complexes. Given the similar impact of both PKA and PHB complex on mitochondrial dynamics, cristae structure and OXPHOS, it would be interesting to examine whether mt-PKA affects PHBs phosphorylation and functions. Such works would clearly improve our understanding of how PHB interactors are involved in mt-PKA signaling and mitochondrial physiology.

Our in silico analyses identified TIM44-S80 as a potential target of PKA. Interestingly, overexpression of TIM44 alone partly rescued the alteration of respiration by mt-PKA, which could indicate that mt-PKA affects OXPHOS via TIM44. However, both the phosphomimetic and the phosphodeficient of TIM44-S80 reversed the mt-PKA-dependent decrease of respiration, suggesting that phosphorylation of TIM-S80 is not involved in the effect of mt-PKA. It is nevertheless possible that the substitution of serine for aspartic acid was not optimal to mimic the phosphorylation of S80 in terms of charge and protein conformation. A better characterization of the TIM44-S80 phosphomimetic should be important to address the role of PKA-TIM44 signaling in future studies.

TIM44 is a peripheral IMM protein interacting with the matrix face of the TIM17/23 translocon [85]. TIM44 recruits different motor proteins, including the mitochondrial Hsp70 (mtHsp70) and Pam16 to the translocon to allow proper import of proteins in the mitochondrial matrix [86]. TIM44-S80 is located at the N-terminal domain which is known to bind TIM17/23, mtHsp70 and Pam16 [86,87] and serves as a dynamic arm to drive efficient translocation of matrix proteins [88], suggesting that phosphorylation of TIM44-S80 by PKA could induce conformational changes of the scaffold TIM44 and impact on import of matrix proteins. We did not observe alterations in the levels of the matrix protein SOD2 or the TIM44 interactors Hsp70 and Pam16 upon expression of TIM44-S80 phosphomutants. Although these results suggest that phosphorylation of TIM44-S80 do not impact on the stability of matrix proteins, it will be important in future studies to examine the impact of (de)phosphorylation of TIM44-S80 on its interaction with the motor proteins mtHsp70 and Pam16 and in the regulation of mitochondrial protein translocation across the IMM.

Among the targets of PKA previously identified, only AKAP1 and MIC19 were confirmed by our work. AKAP1 anchors PKA to mitochondrial membranes [16,23], whereas PKA-mediated phosphorylation of MIC19 negatively regulates mitophagy by impairing Parkin recruitment to damaged mitochondria [89]. Future studies should examine whether such mechanisms could have been involved in the PKA-mediated alterations of ROS levels and cellular respiration observed here. Numerous other known PKA substrates, as described above, were however not identified by our approach. The criteria that we used to filter MS data could have led to elimination of PKA substrates previously identified. For instance, AIF was detected by LC-MS/MS but was discarded since there was not a difference of five detected peptides between cells expressing pcDNA and mt-PKA-BioID2-HA. Several other components of OXPHOS were detected (Tables S1–S5) but were also discarded according to our filters. It is thus possible that these proteins could be involved in the mt-PKA-mediated effects on mitochondrial activity.

Our BioID approach does not discard the possibility that the mt-PKA-BioID construct interacted with the identified interactors, such as TIM44-S80, outside of the mitochondrial matrix. For instance, the OMM proteins Drp1 and TOM70 were identified as potential targets of PKA. PKA phosphorylates Drp1-S637 and induce detachment of Drp1 from OMM and inhibition of mitochondrial fission [90]. TOM70 is part of the translocase of the outer membrane complex and recognizes preproteins with hydrophobic domains destined to the IMM [91,92]. Moreover, several non-mitochondrial proteins were detected by our BioID assay (Table S4). In fact, the mt-PKA-BioID2 constructs targeted 208 proteins, among which 33 are mitochondrial proteins (Tables S3 and S4). These findings suggest that our BioID2 construct likely interacted with several extra-mitochondrial proteins before its complete translocation within the organelle. The interactomes of the different mitochondrial subcompartments were recently characterized using BioID2. Similar to our findings, this work identified a total of 1465 proteins, among which only 528 were mitochondrial proteins [93]. Considering that BioID2 is constitutively active and that cells are treated with biotin during 24 h, it is thus possible that BioID2 constructs identify interactors from the site of synthesis to their final destination, as previously discussed [94,95]. Recent development in proximity biotinylation techniques could help to circumvent these biases. For instance, the TurboID enables shorter biotin treatment [96] whereas 2c-BioID allows to keep the protein of interest separated from the biotin ligase until the biotin treatment [94].

## 9. Conclusions

Taken together, our results demonstrate that the overexpression of mt-PKA decreases mitochondrial respiration and enhances ROS levels. We also identified various mitochondrial proteins involved in metabolism and stress response as potential targets of mt-PKA. It will be important in future studies to examine whether the 21 targets of the mt-PKA-BioID2 construct are phosphorylated by endogenous PKA. It will also be important to verify whether intramitochondrial phosphatases, such as Pptc7 [97], PGAM5 [98,99], PP2B [33,100] and PPM1K [101], are able to counteract PKA-mediated phosphorylation of these proteins. These works will provide insights about potential new signaling pathways triggered and regulated inside mitochondria.

## Figures and Tables

**Table 1:** Potential mitochondrial targets of PKA located inside mitochondria. Twenty-one potential interactors were identified by BioID2 coupled to LC-MS/MS.

Protein Name	Accession Number	Predicted Phosphoresidue	Molecular Function
AKAP1	Q92667	S107, S191, S235, S577	Kinase anchoring
DRP1	G8JLD5	T79, S637	Mitochondrial shape
TOM70	O94826	S81, S375	Protein import
TIM44	O43615	S80	
Hsp60	P10809	S159	Protein folding and stress response
TRAP1	Q12931	S180	
TCP-1-eta	Q99832	T526	
CLPX	O76031	S220	Protein degradation
IleRS	Q9NSE4	S818	tRNA maturation and mRNA metabolism
TRMT10C	Q7L0Y3	T382	
LRP130	P42704	S1022, S1302, S1393	
RNS4I	P61221	S560	
Citrin	Q9UJS0	S469	Metabolites exchange
P5CR1	P32322	S109	Response to oxidative stress
SHMT2	P34897	S184	
GDH1	P00367	T206	TCA cycle regulation
ACL	P53396	S455	
ATAD3A	Q9NVI7	T166, T221	Mitochondrial protein synthesis and maintenance of cristae junctions
MIC19	C9JRZ6	T11, S29, S50	
HAX-1	O00165	S53	Cell survival
MAVS	Q7Z434	S100, S238, S373	

## Supplementary Materials

The following are available online at https://www.mdpi.com/1422-0067/21/21/8283/s1, Figure S1: Supplementary quantification and data, Table S1: Total proteins identified by BioID and LC-MS/MS, Table S2: Proteins identified with a minimum of 5 peptides, a protein false discovery rate (FDR) threshold of 1% and a peptide FDR threshold of 95%, Table S3: Proteins identified with a minimum difference of five peptides between cells expressing pcDNA and mt-PKA-BioID2-HA, Table S4: Mitochondrial proteins identified with a minimum difference of five peptides between cells expressing pcDNA and mt-PKA-BioID2-HA, Table S5: In silico phosphoprediction analysis on mitochondrial proteins interacting with mt-PKA-BioID2-HA.
