# Supplementary material for: Insight into the Interactome of Intramitochondrial PKA Using Biotinylation-Proximity Labeling
Source: Int J Mol Sci. 2020 Nov 5;21(21):8283. doi: 10.3390/ijms21218283 (PMC7663848; doi:10.3390/ijms21218283)

# Original uncropped images

## Figure 1A

PKA Cat

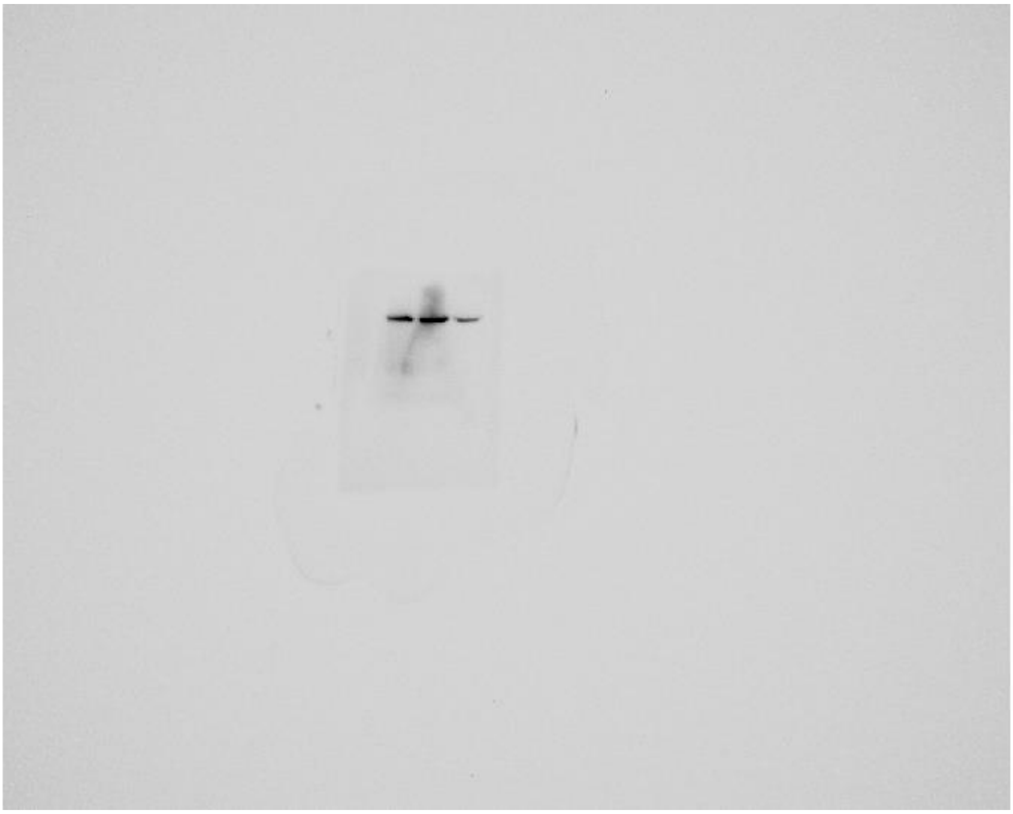

PKA reg

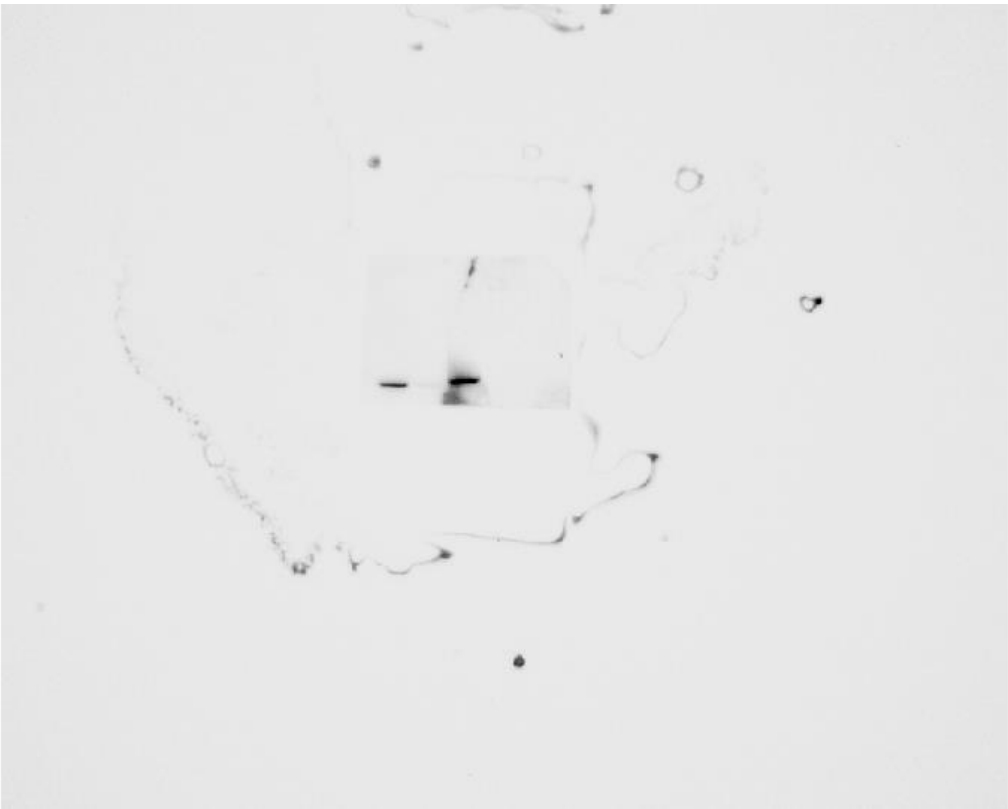

SDHa

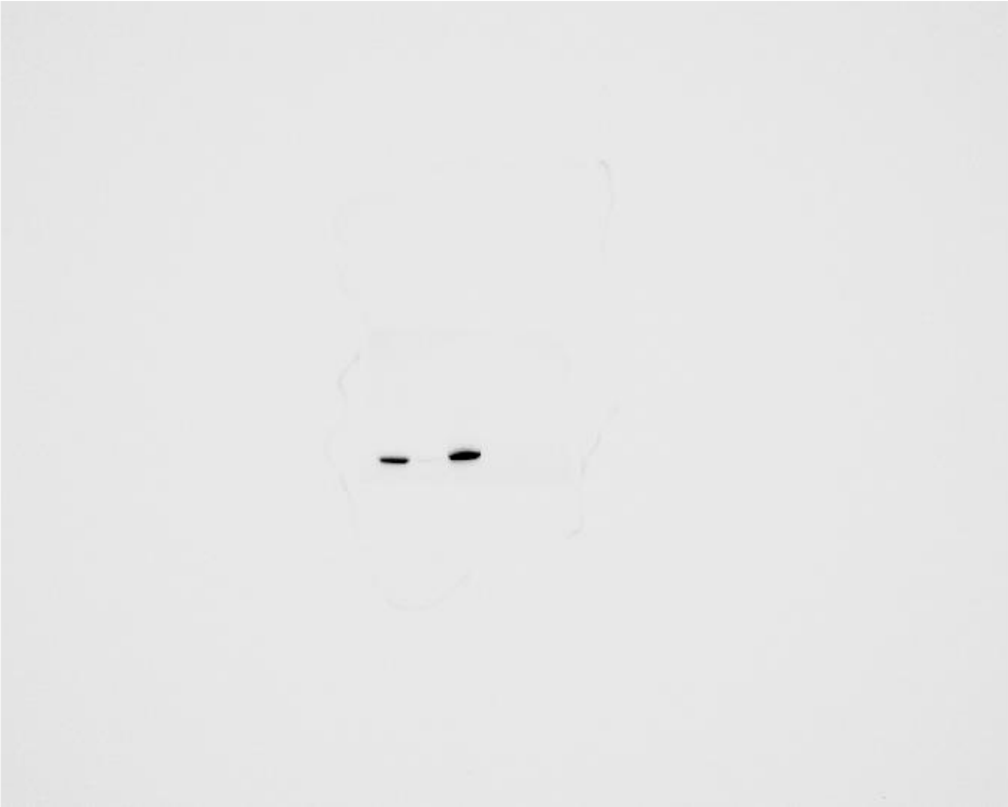

Tubuln

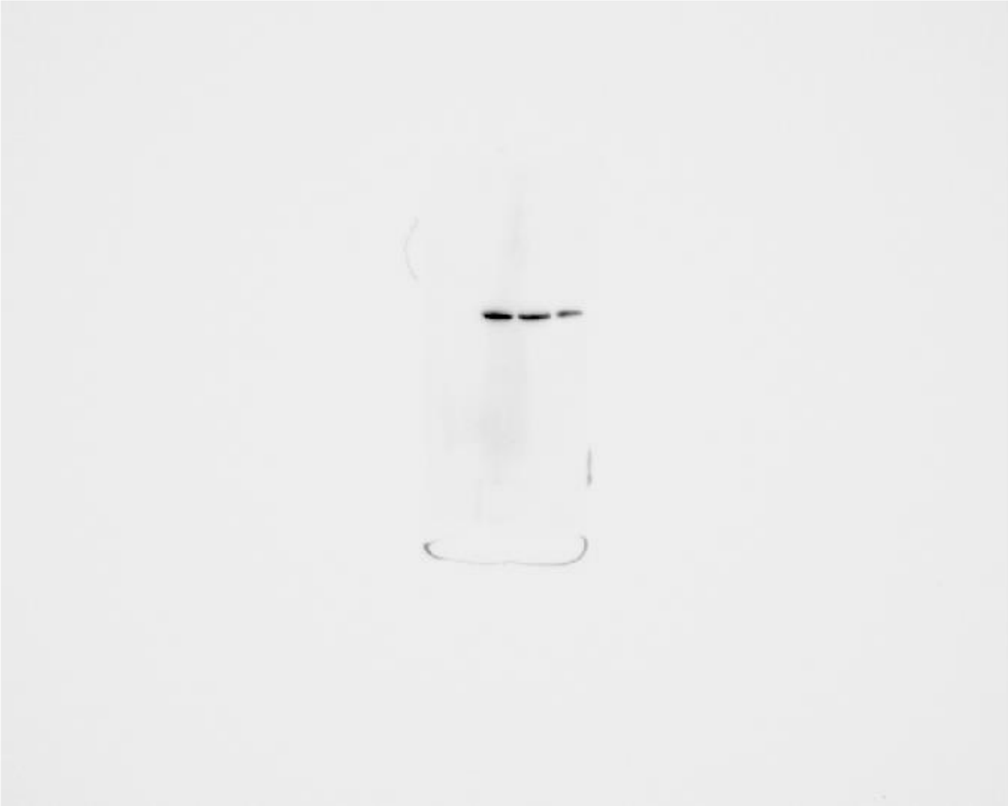

TPL left Fig1A right fig 1B

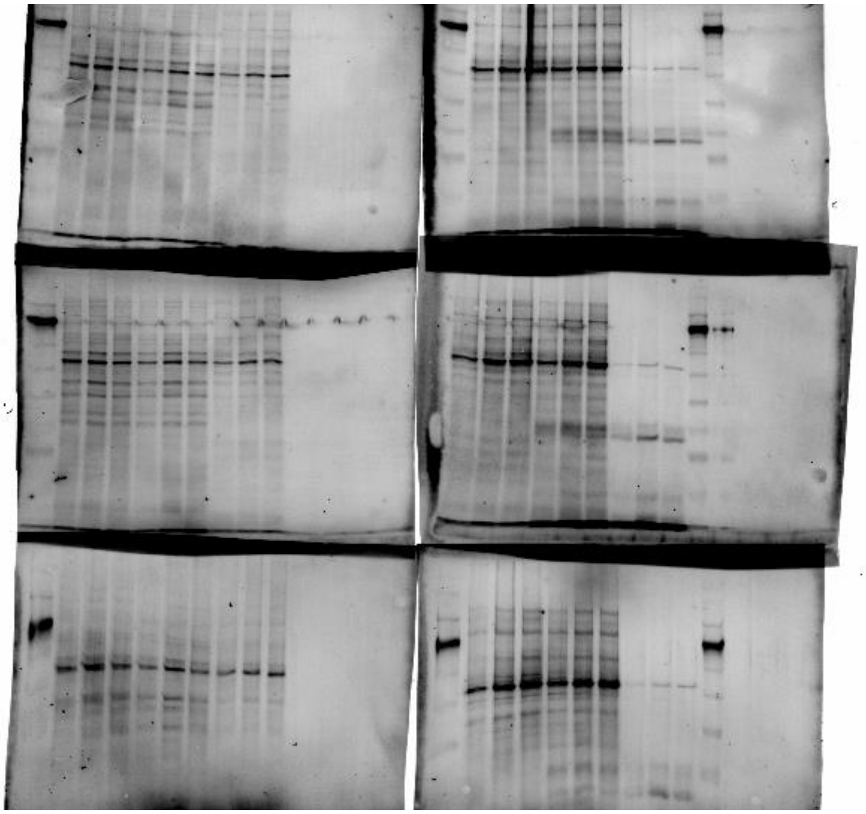

# Figure 1B

PKA Cat

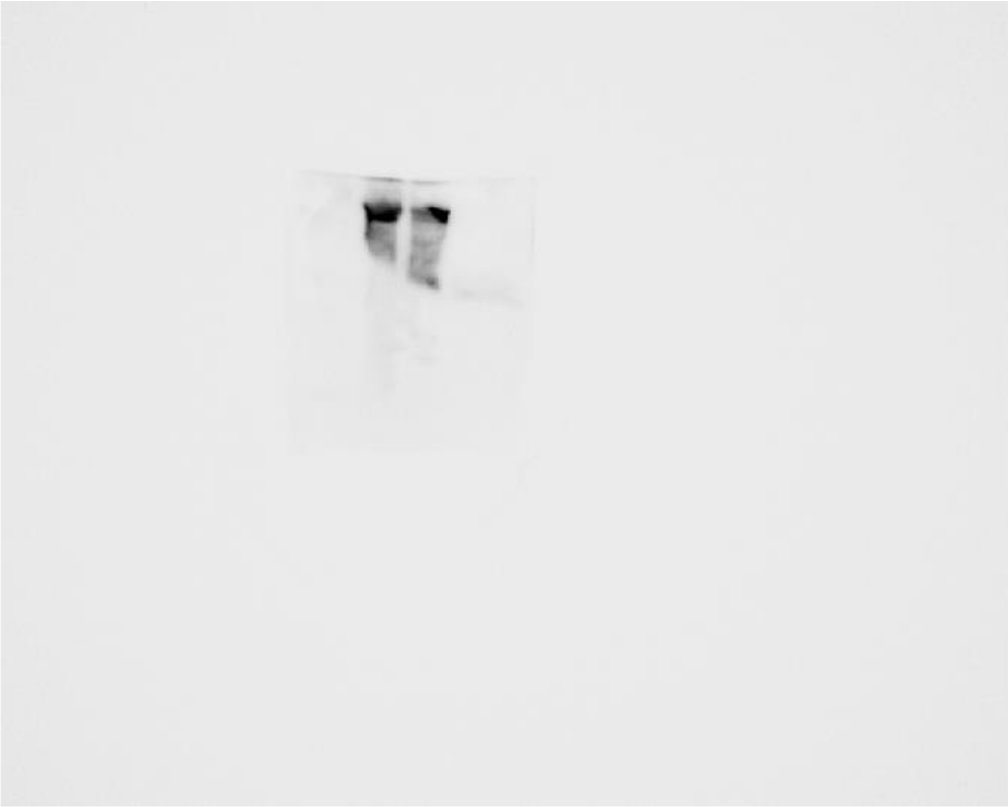

PKA Reg

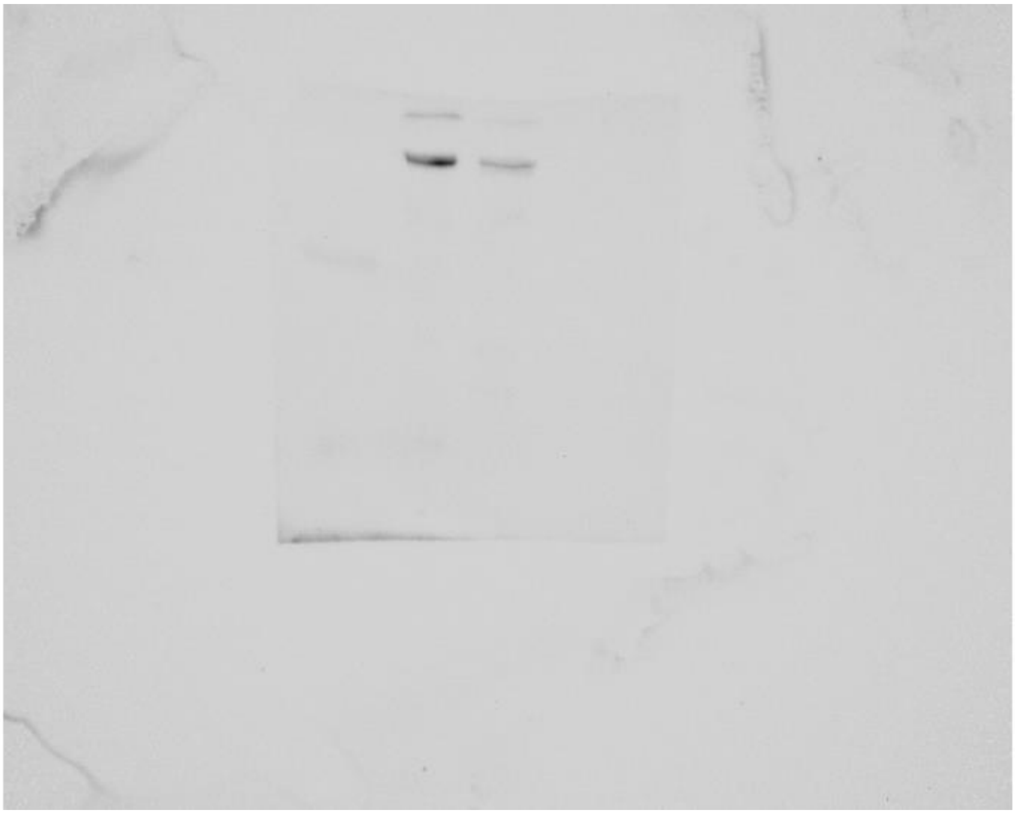

TOM20

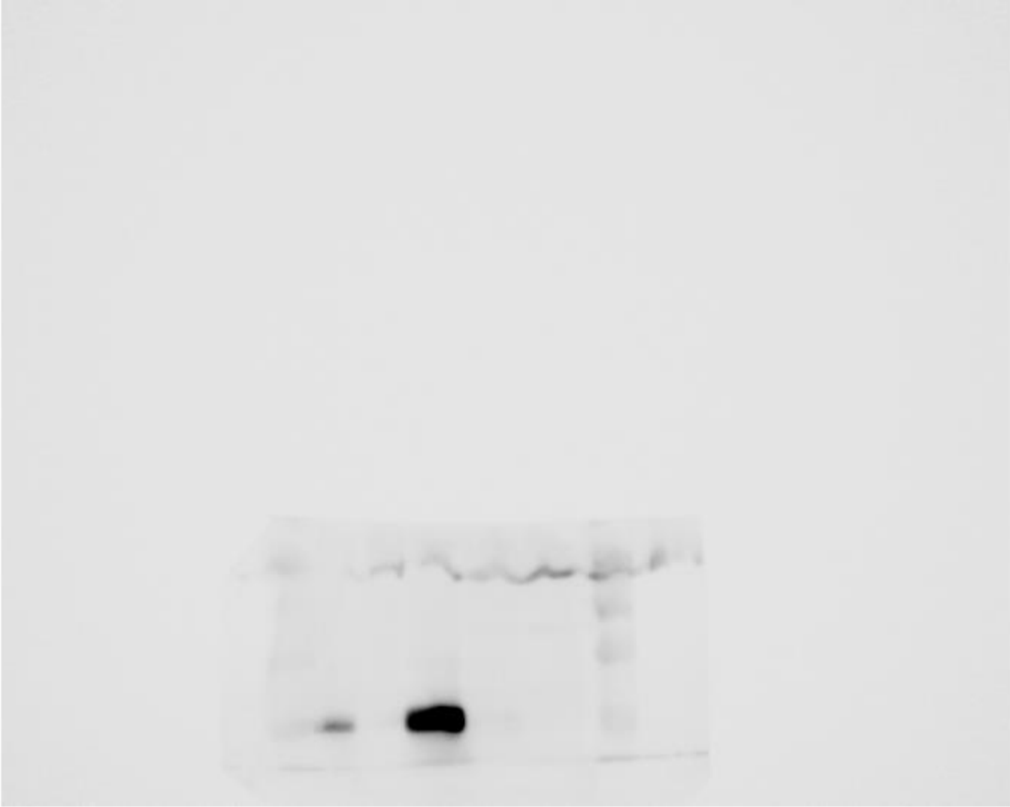

SDHa

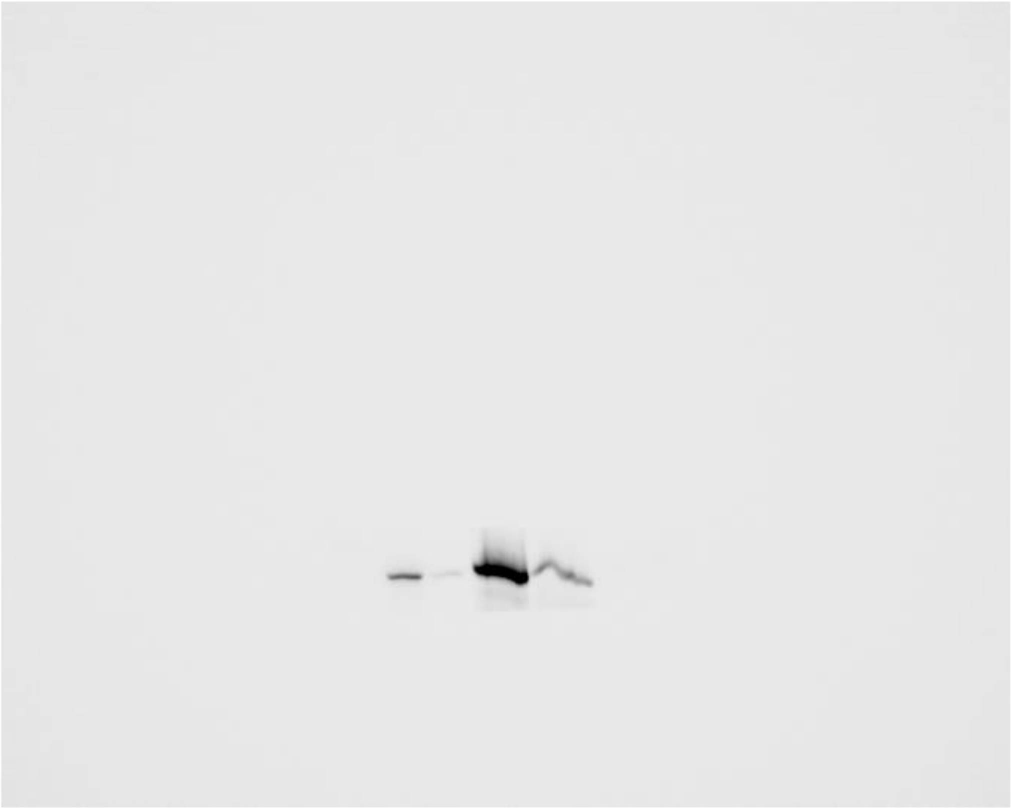

## Figures 1D and 1E

TPL up : TCL-Cyto-Mito /down: Trp sens

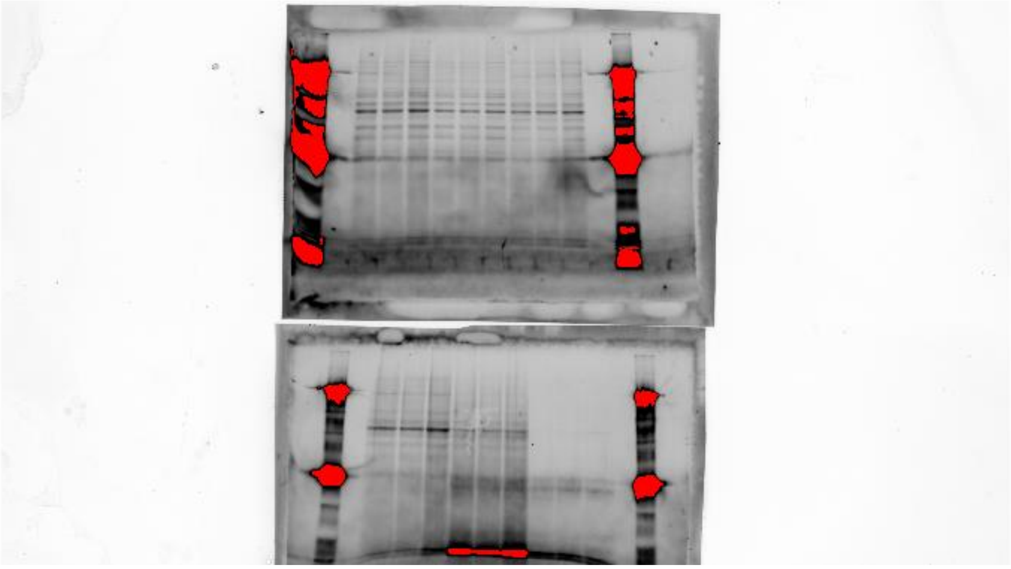

Myc

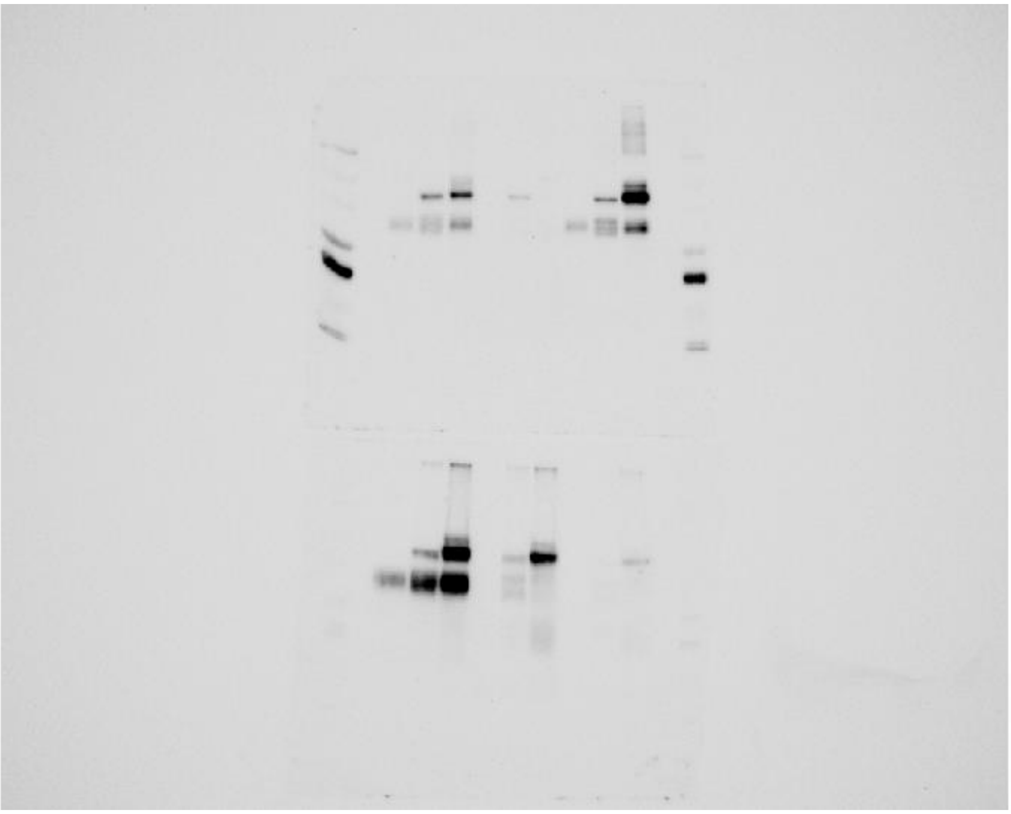

Tubulin  TCL-Cyto-Mito



SDHa TCL-Cyto up and Trp sensitivity assay down

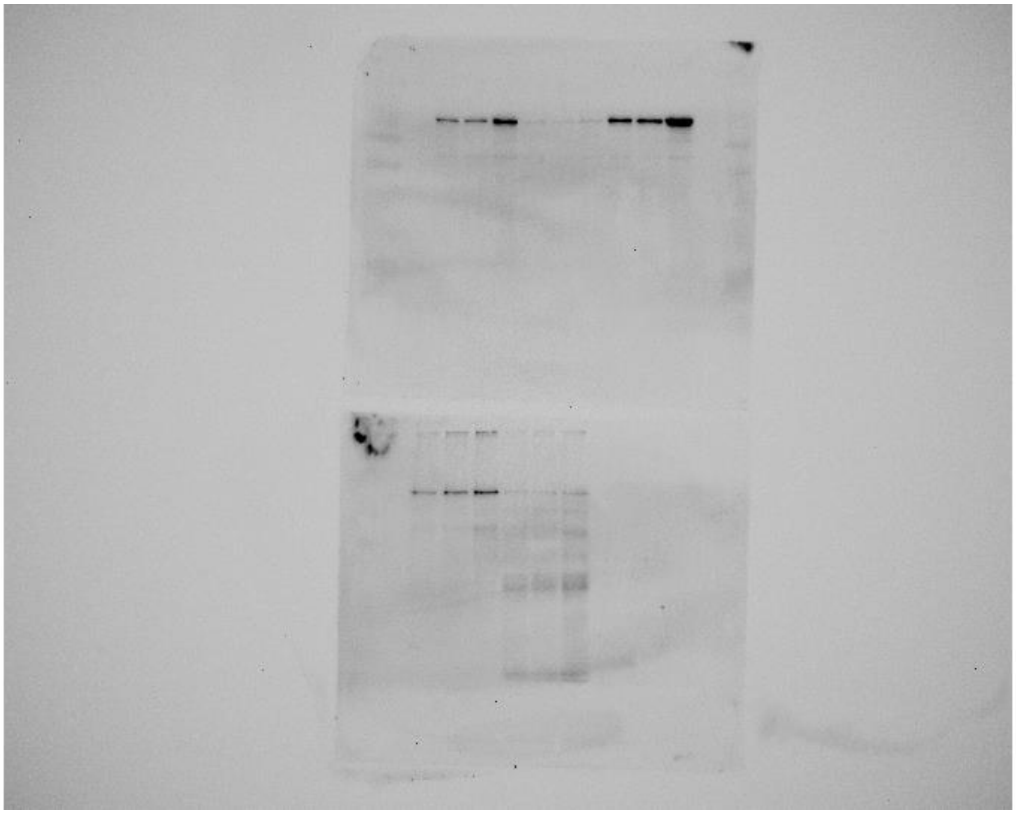

TOM20 Trp

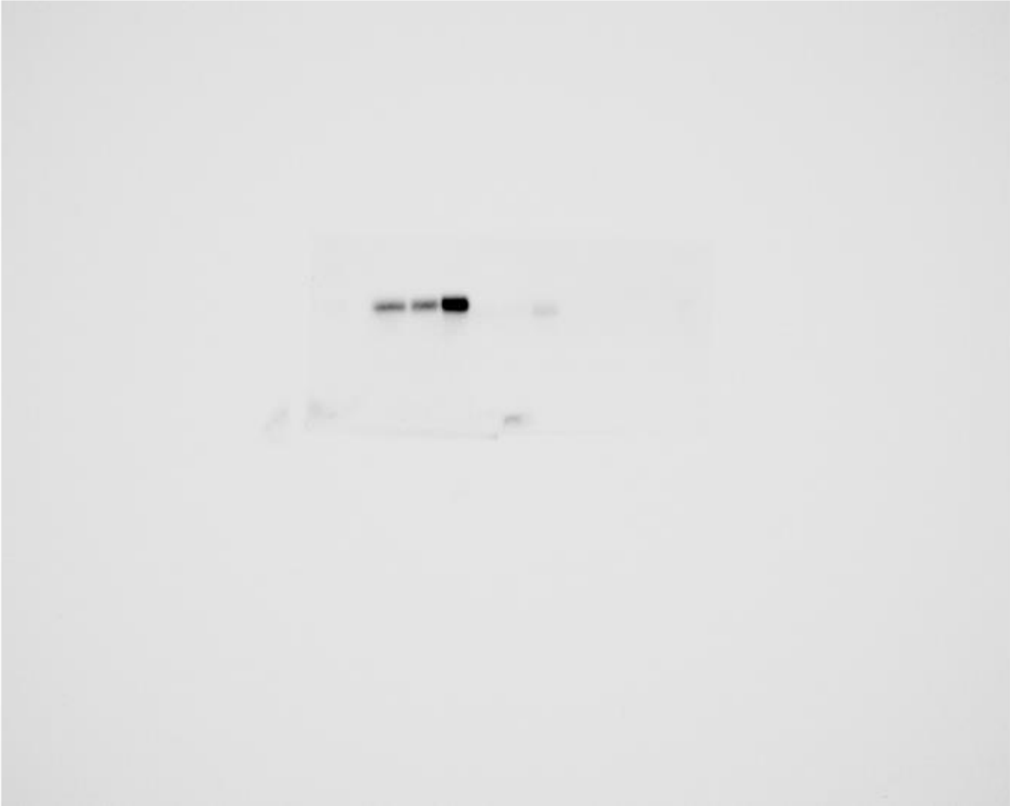

Sod2

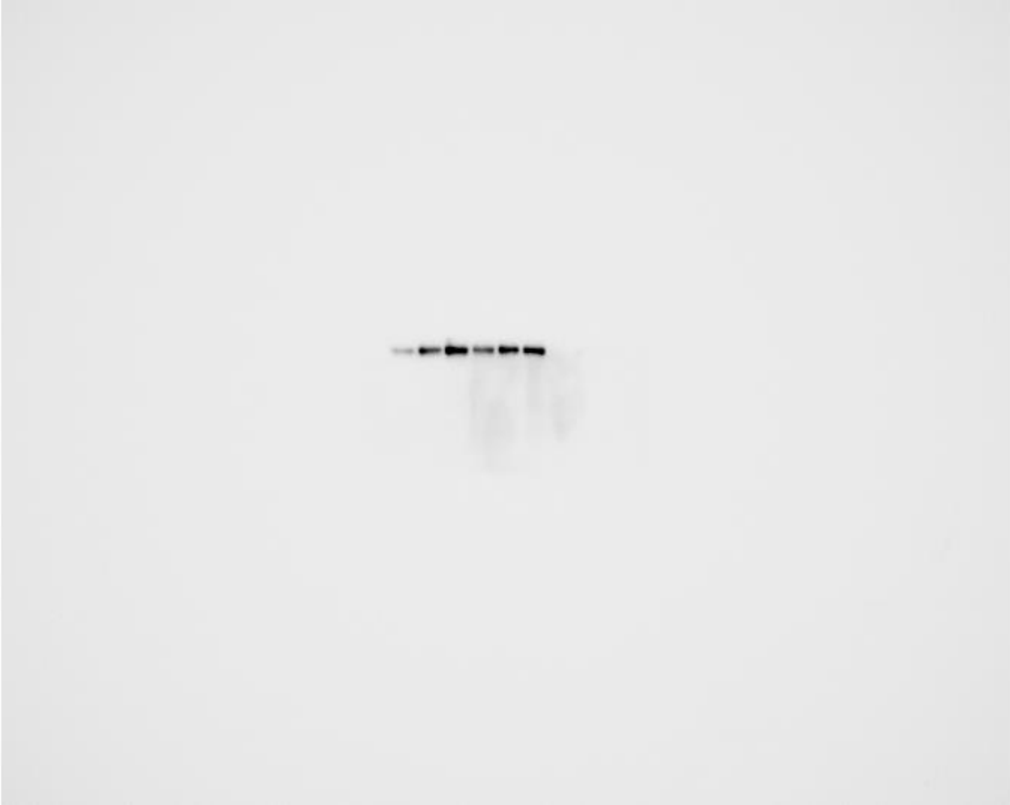

## Figure 1E

Erp57

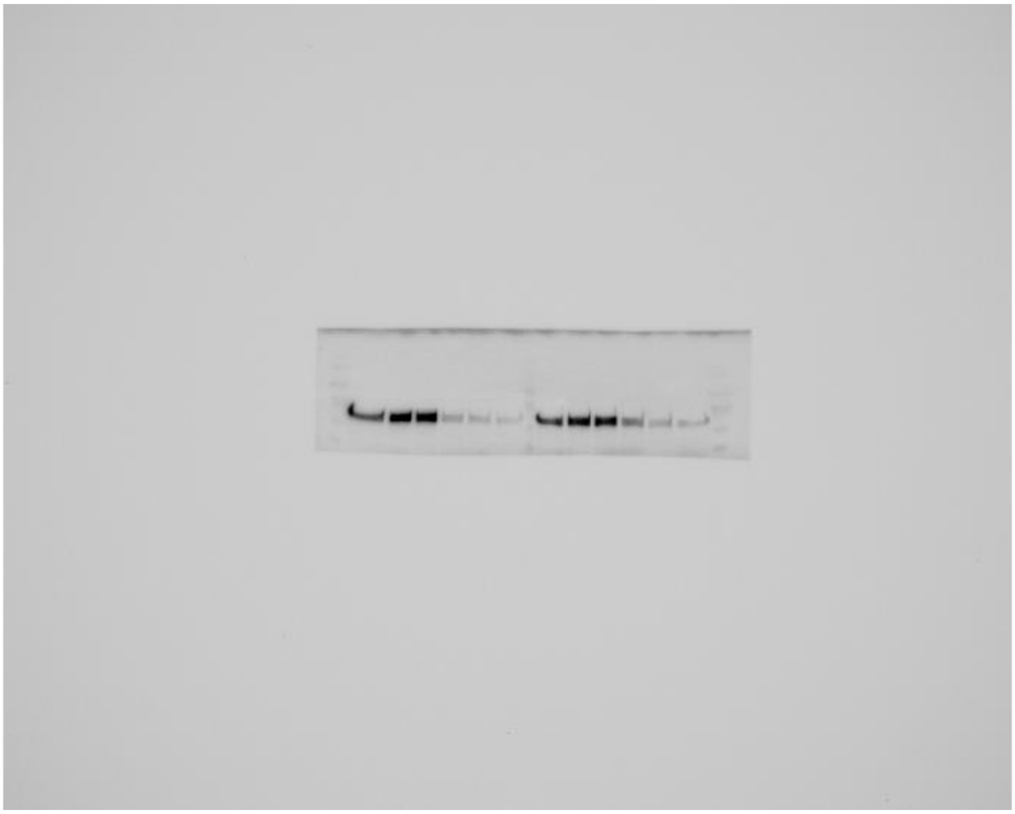

MFn2

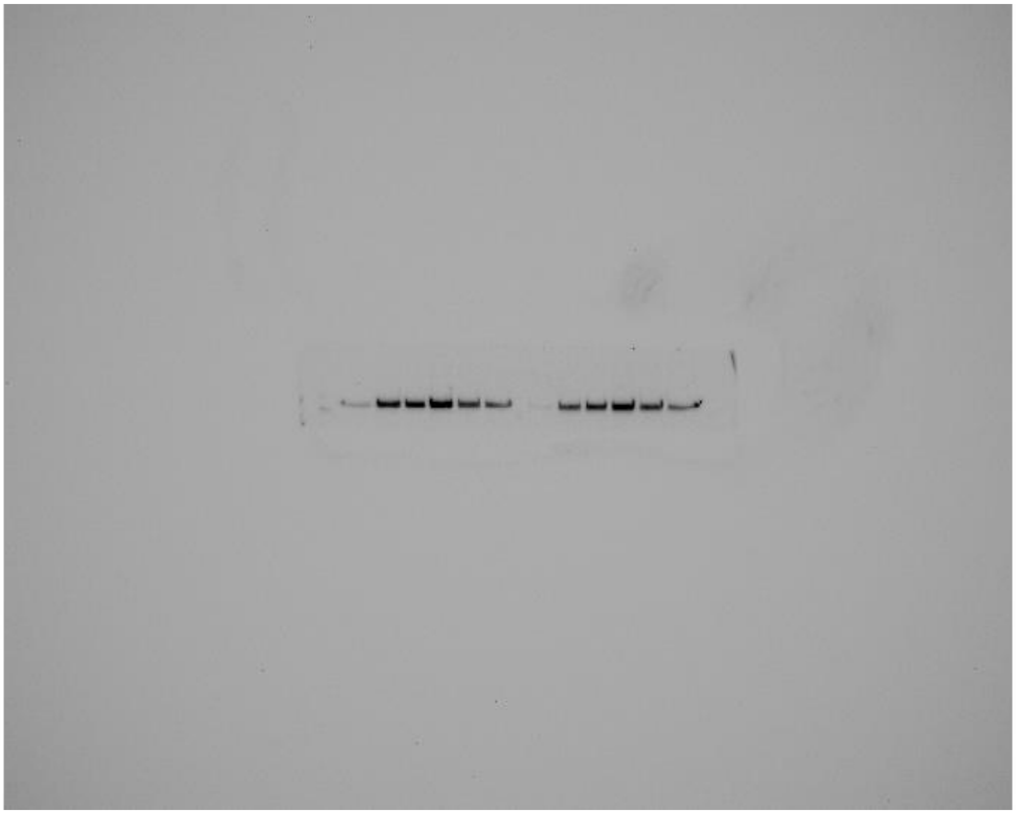

Atp5a

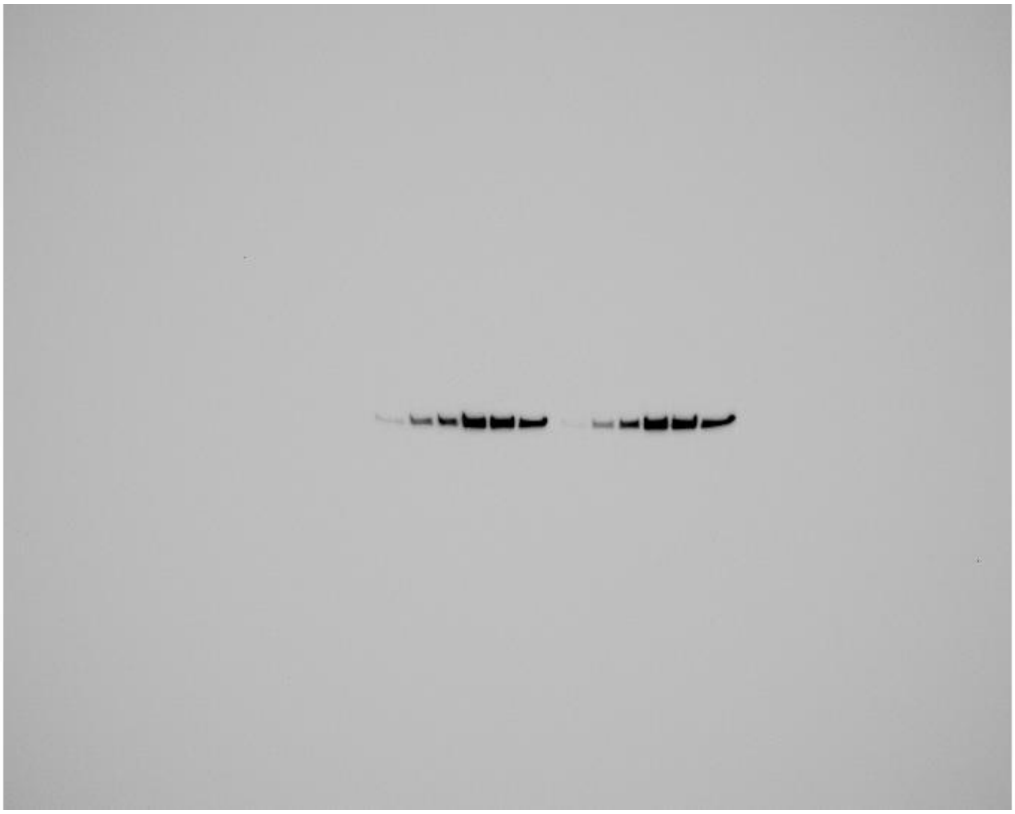

Myc

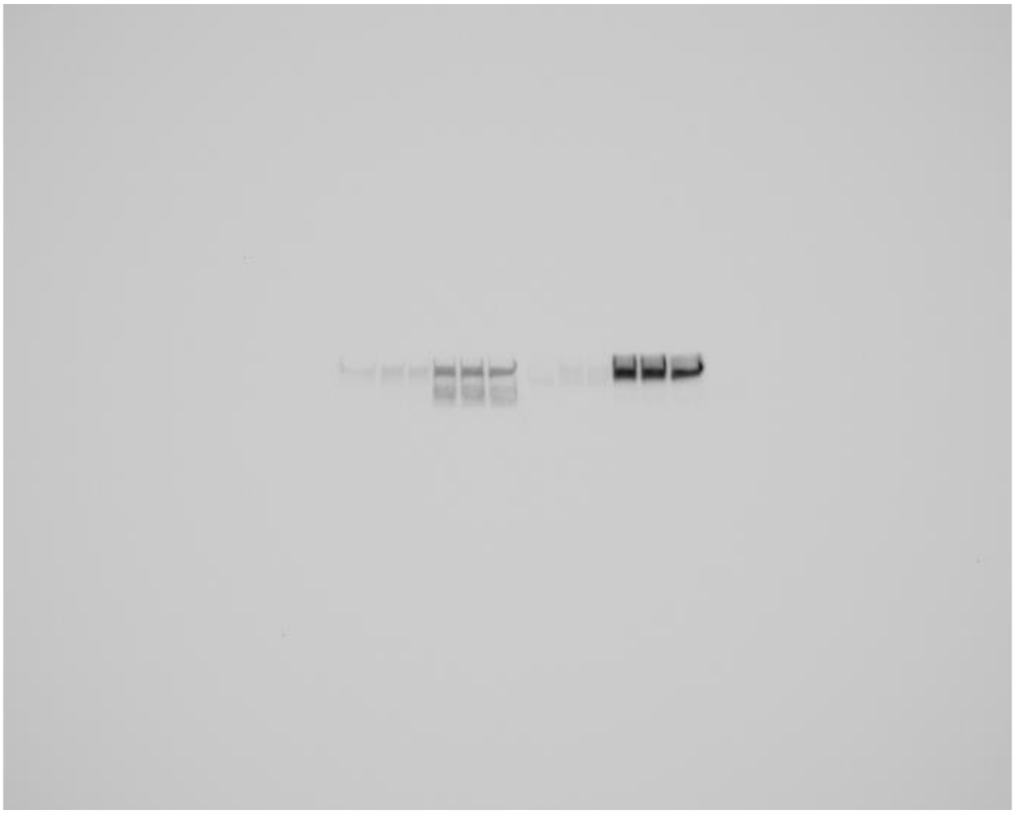

PKA cat

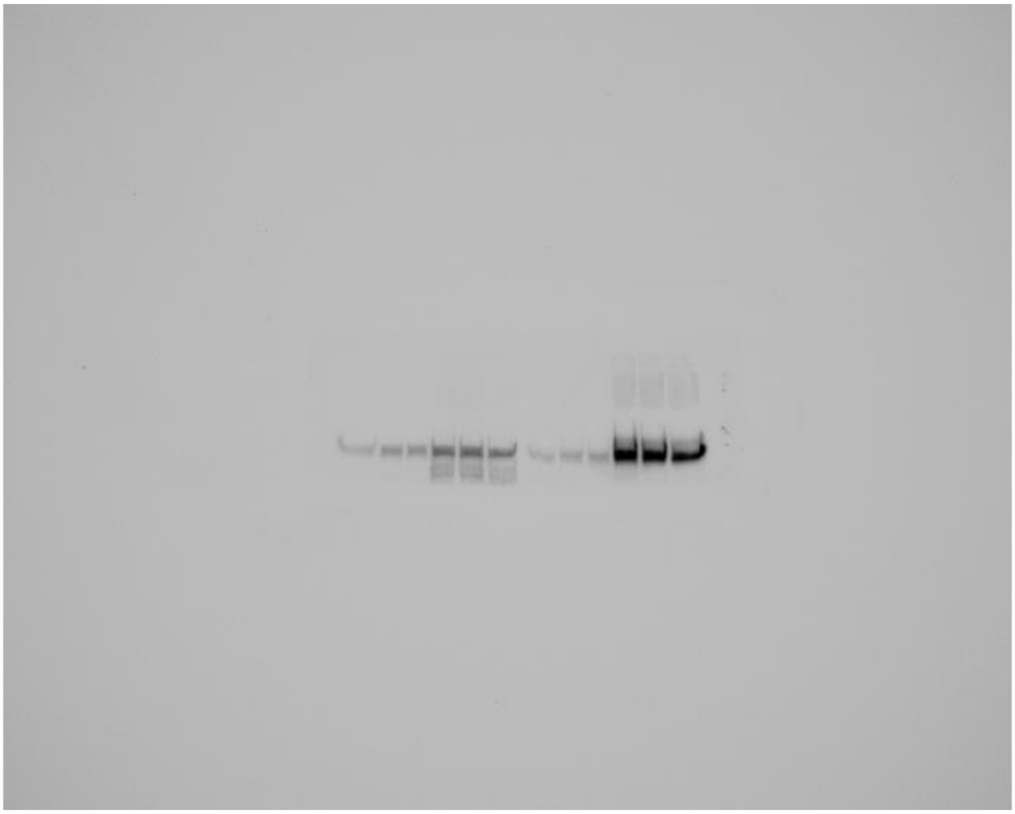

TPL

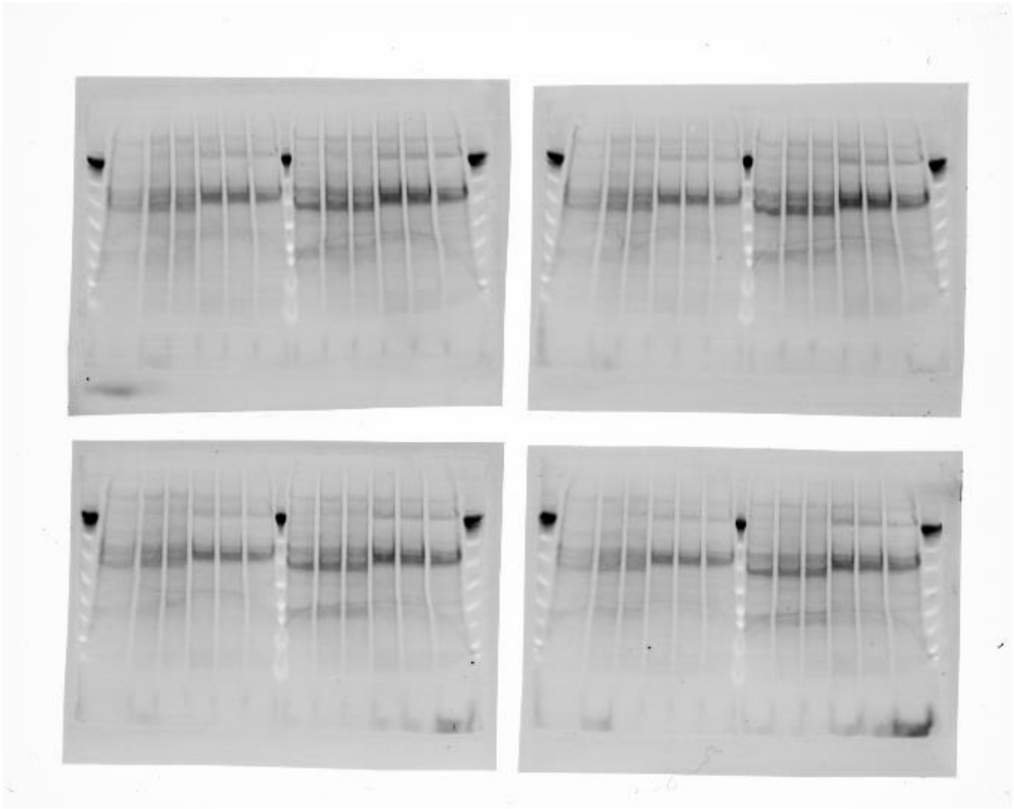

# Figure 1 F

Phospho-PKA substrate TCM

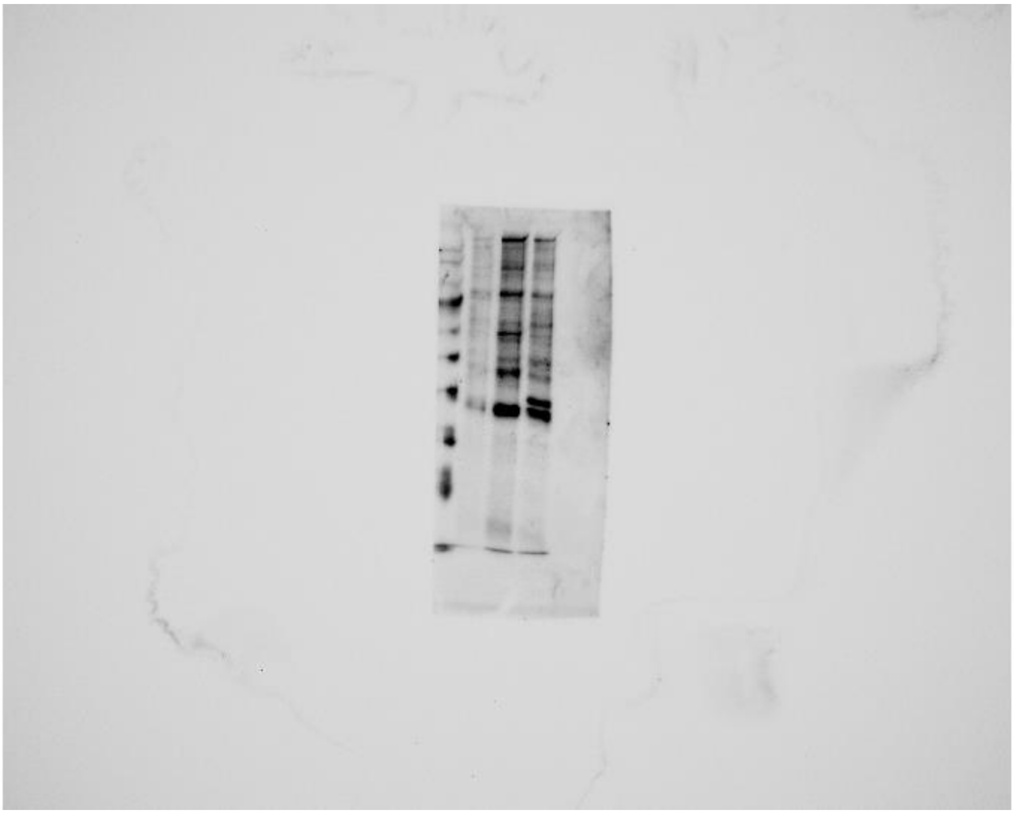

Myc

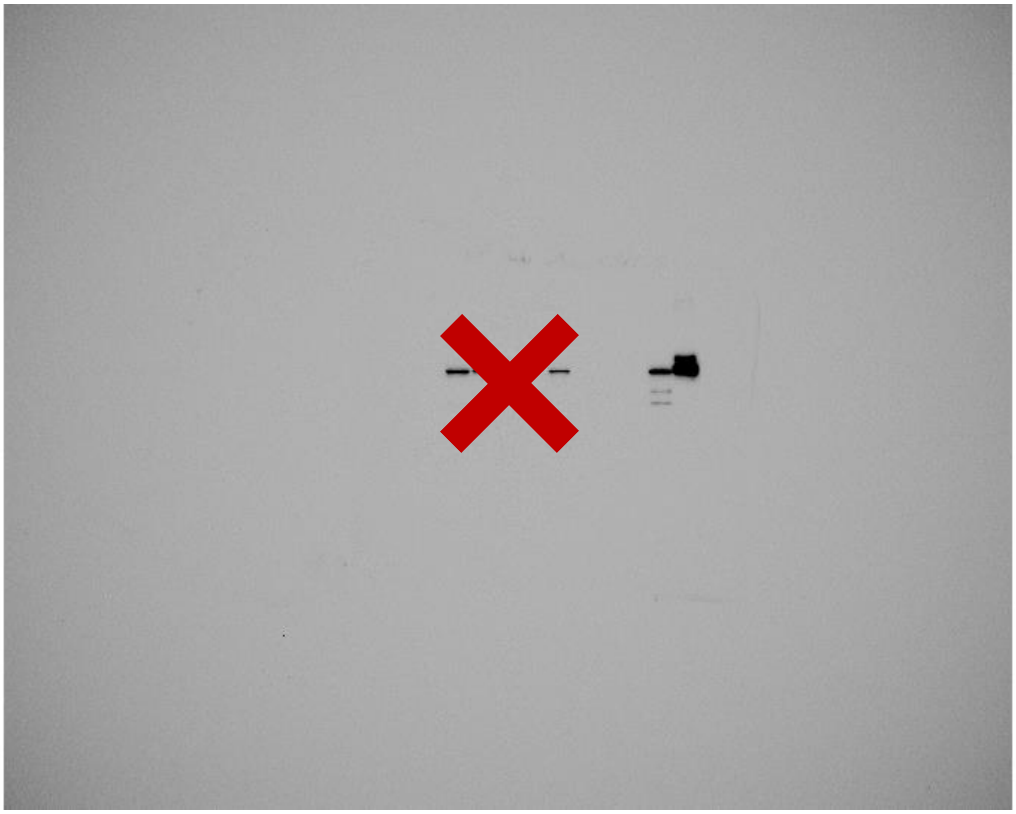

TPL Myc down left and phosphoPKA substarte down right

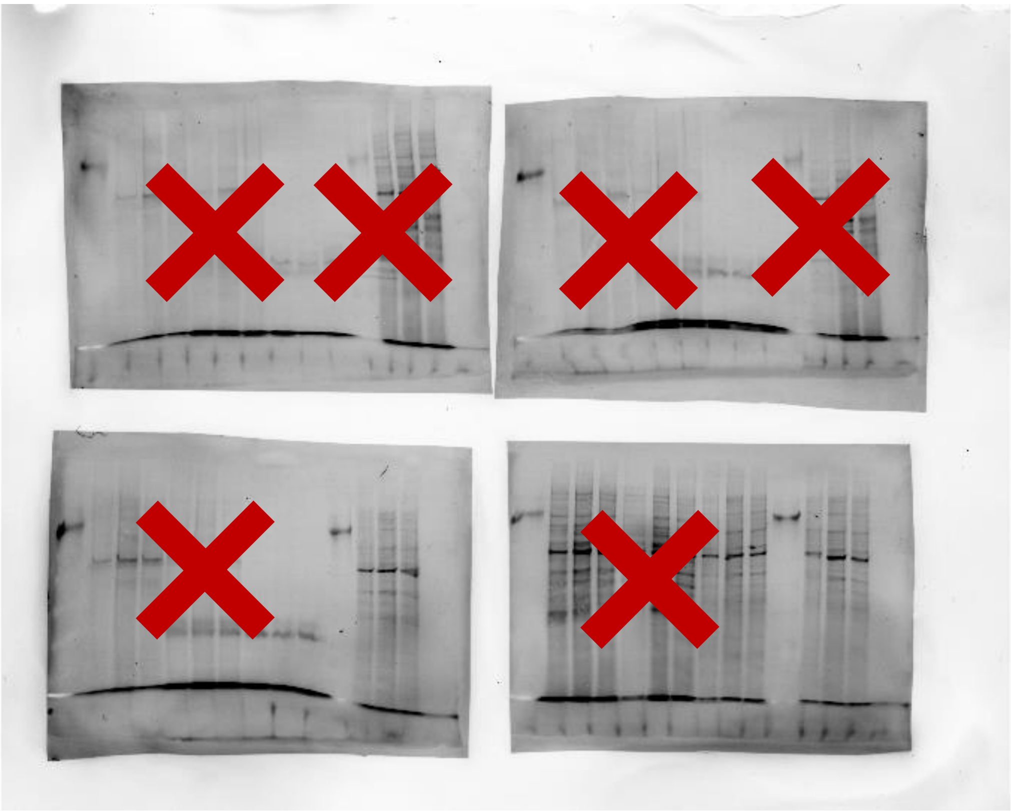

## Figure 3 C

Streptavidin

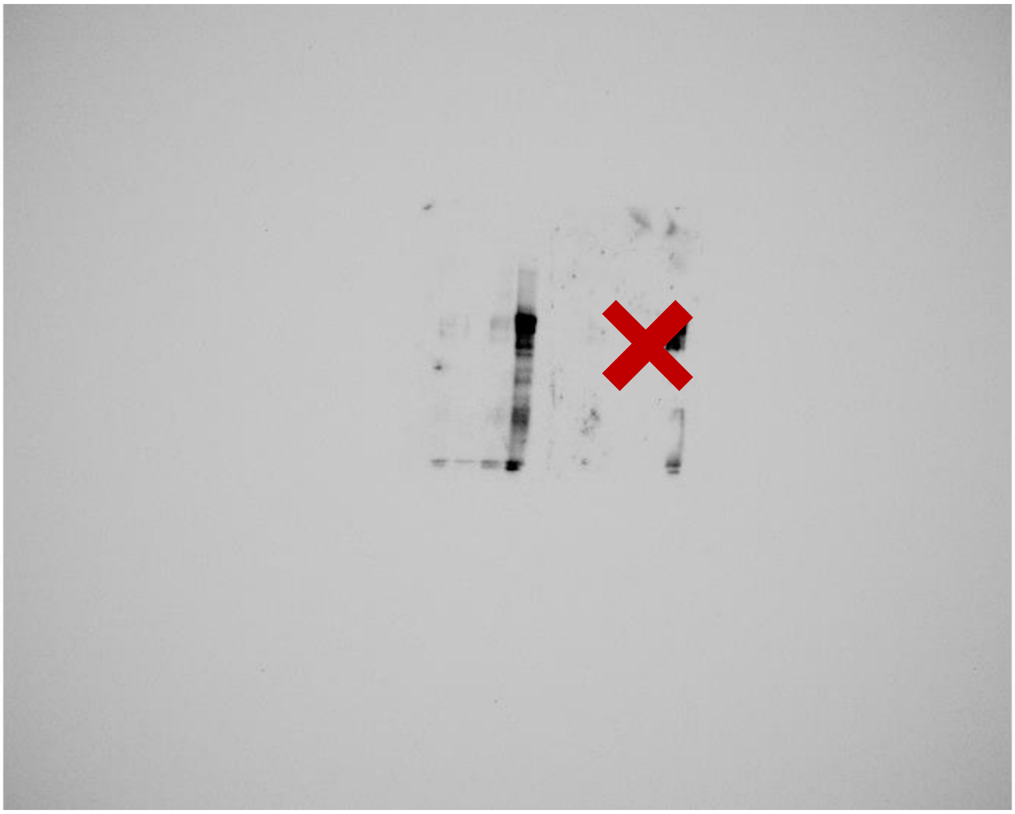

HA right

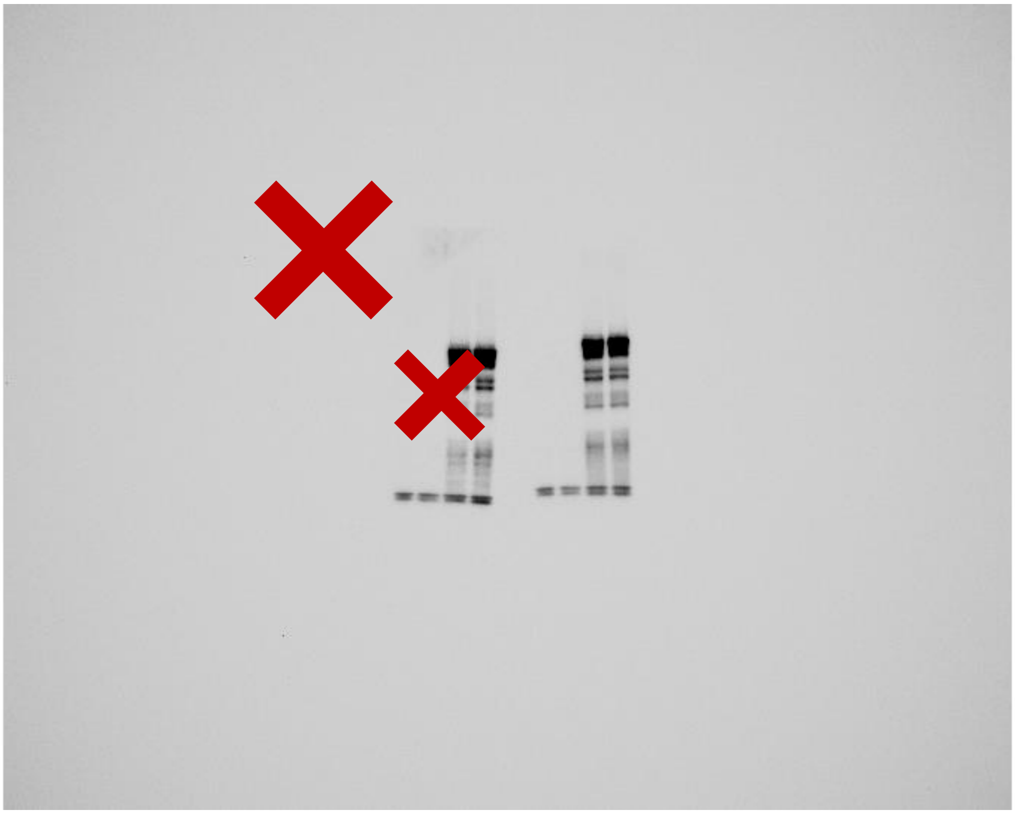

Tubulin right

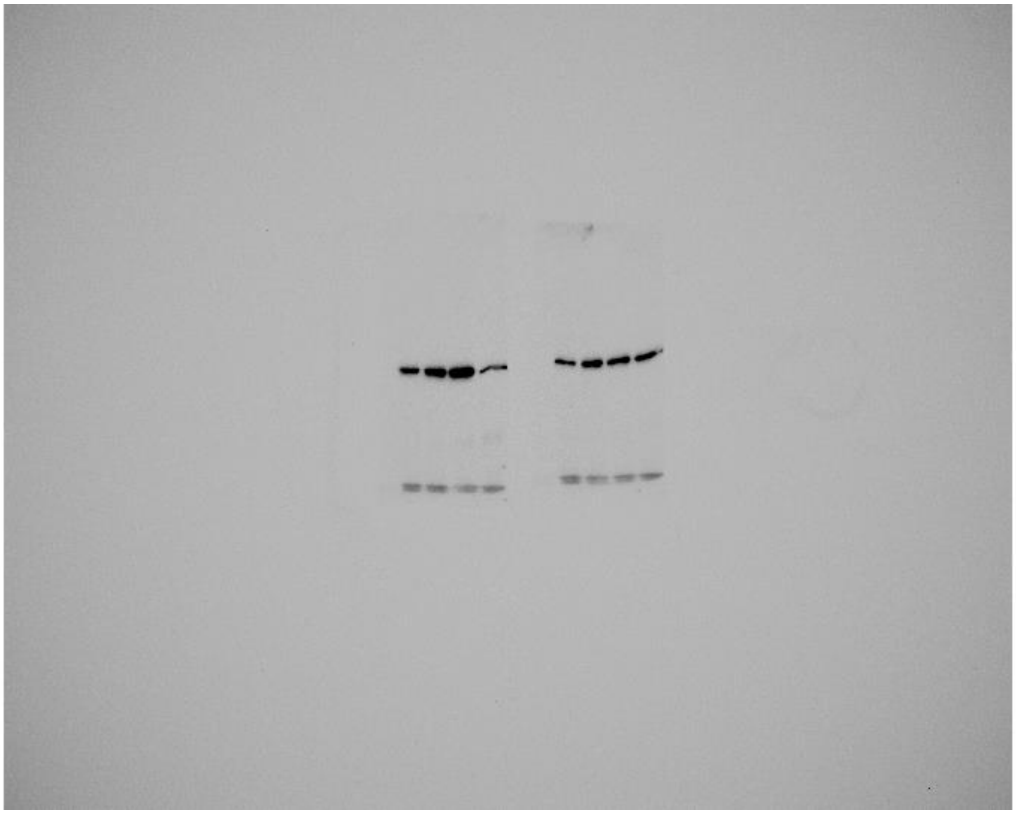

# Figure 3 D

Input V5 for coIP TIM44WT-PKA

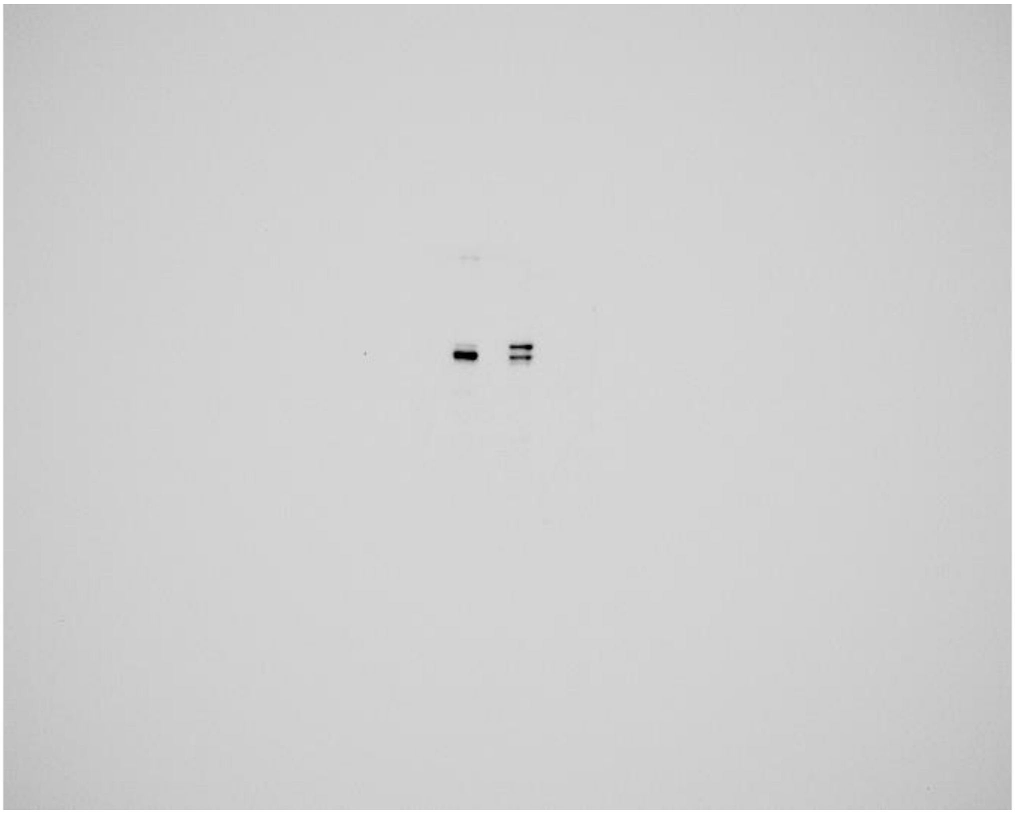

Input Myc

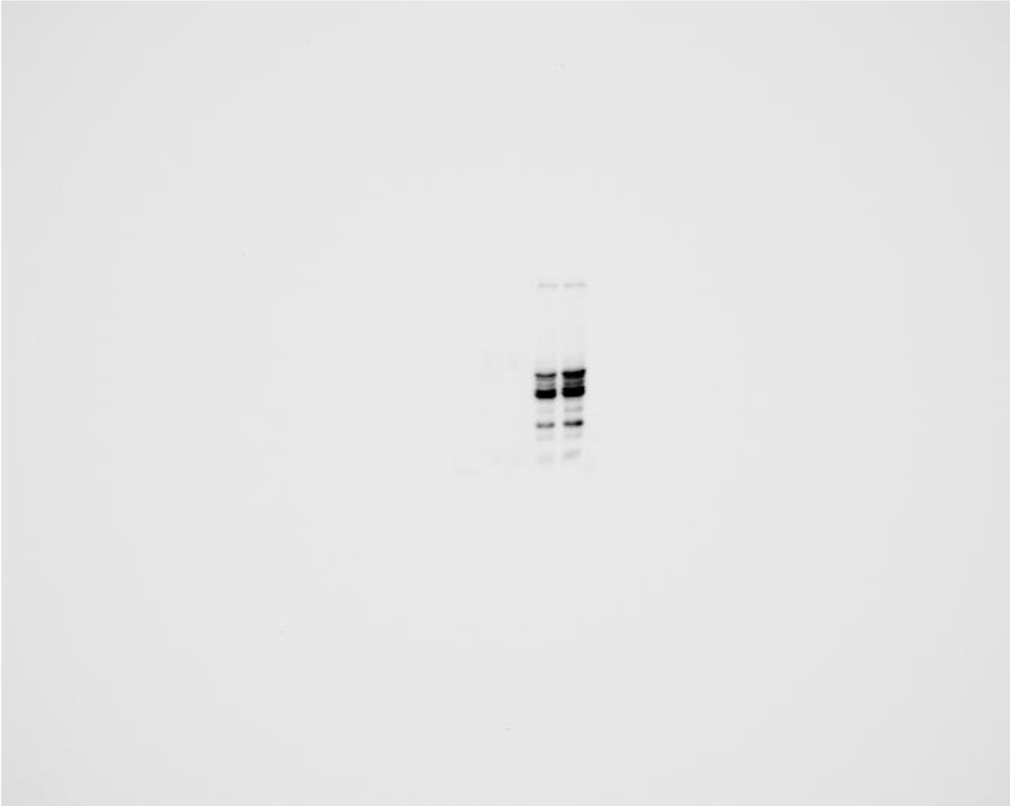

IP V5 anti myc

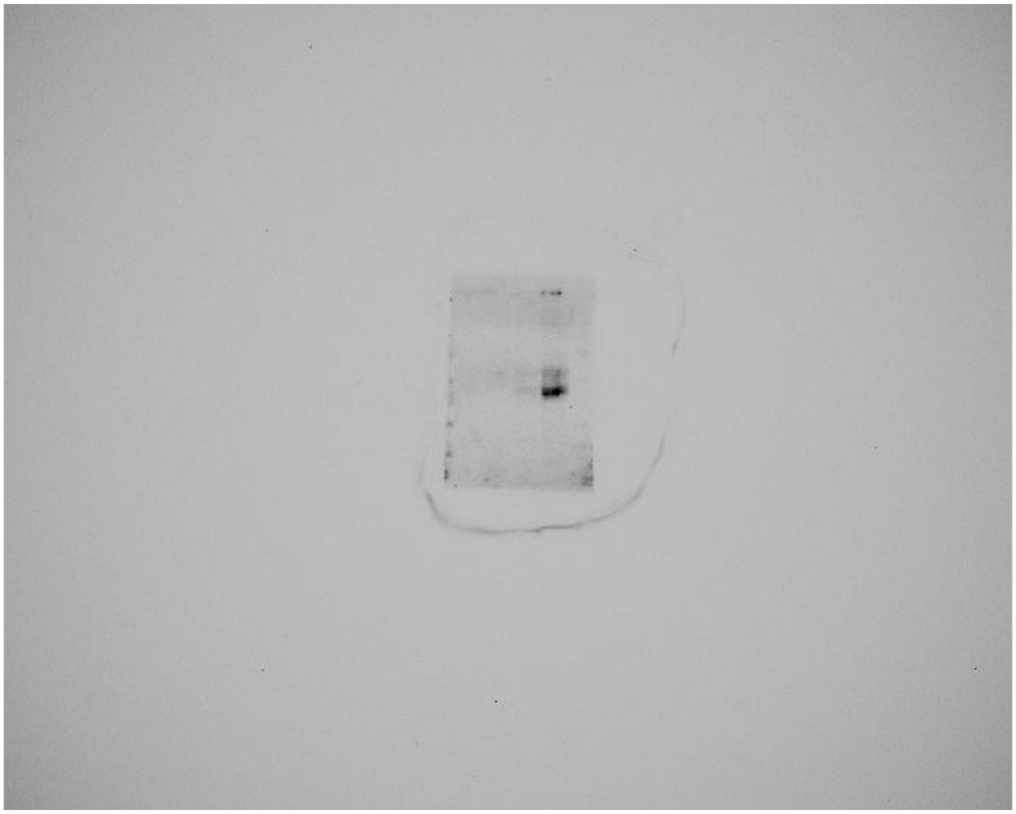

IP V5 anti V5

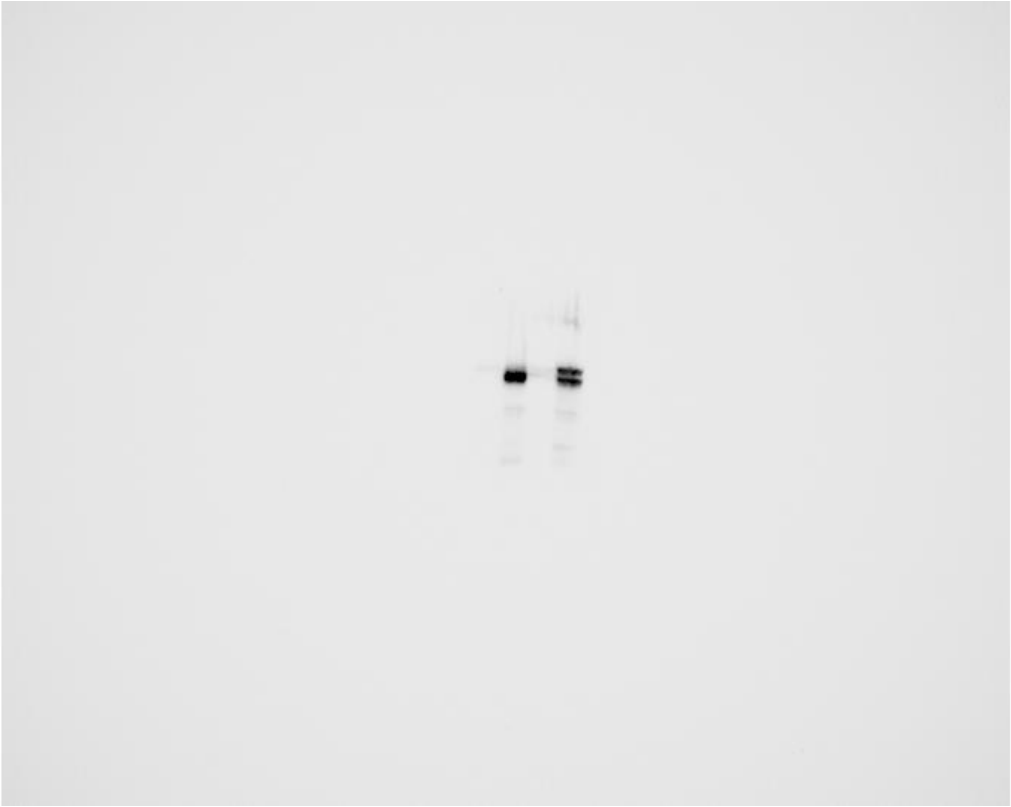

Stain free Input left and IP right

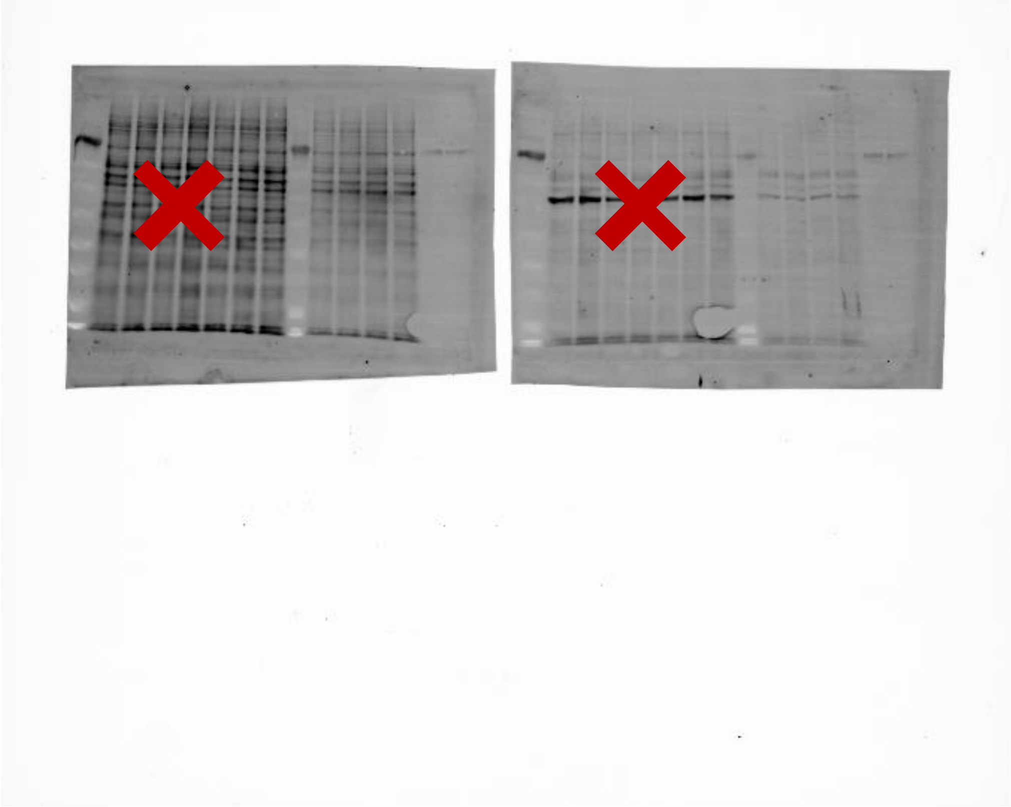

## Fig 4A

TCL TIM44 V5

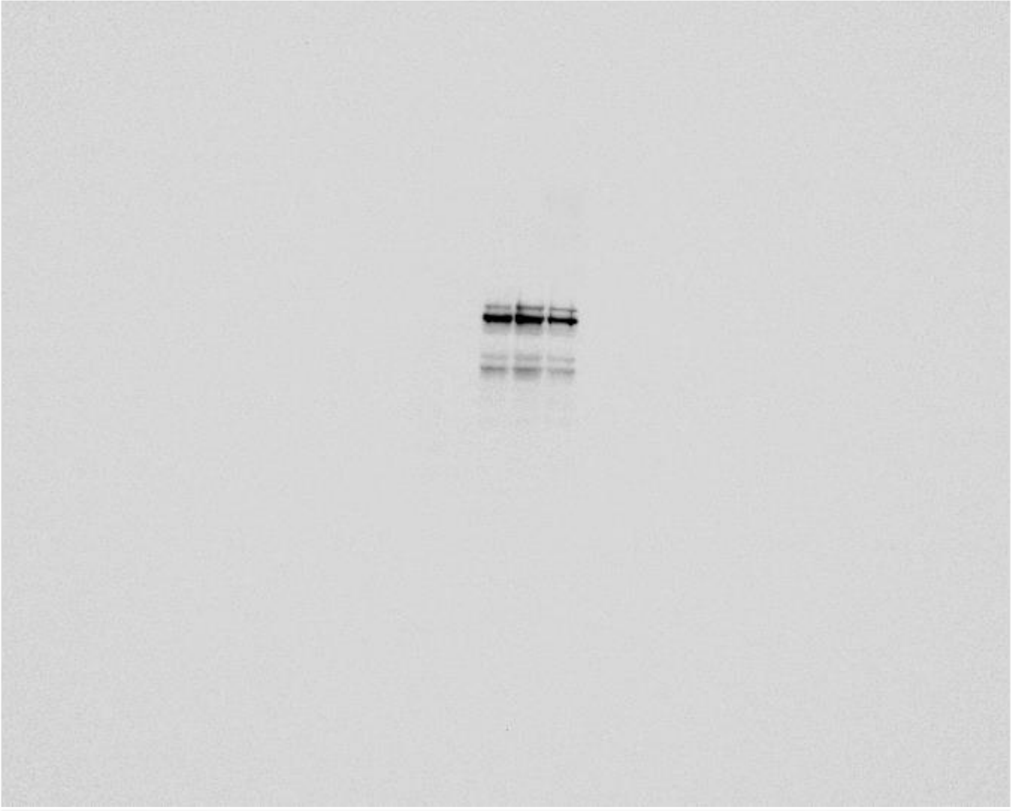

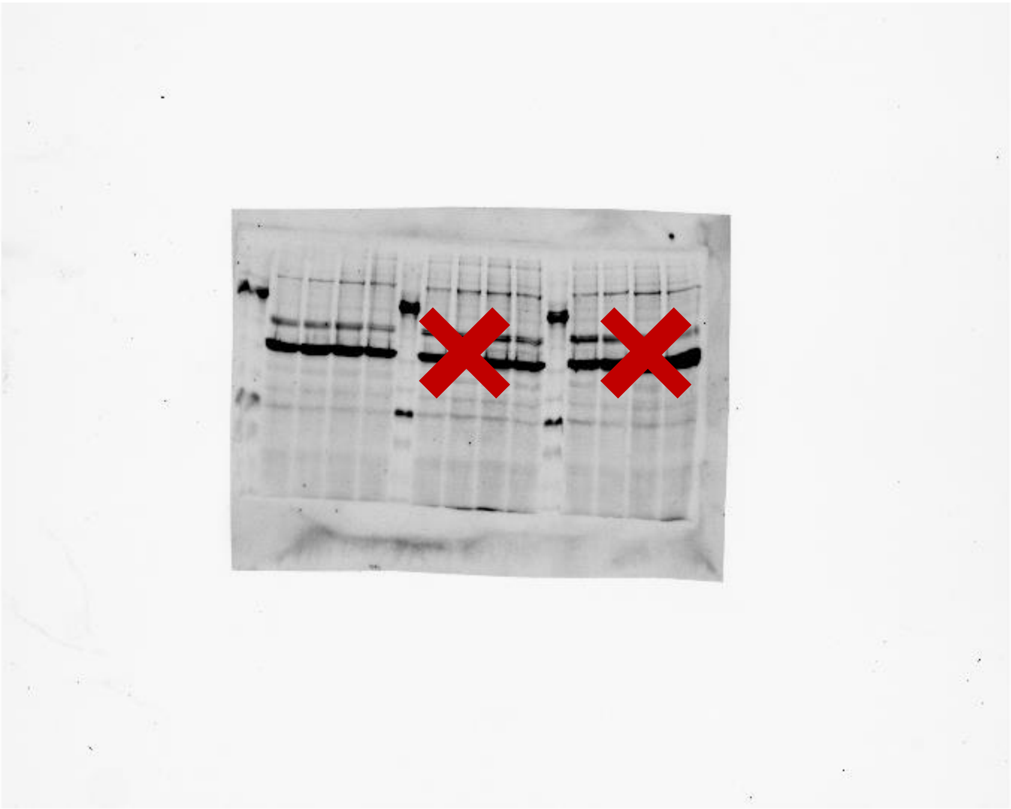

Fig 4B

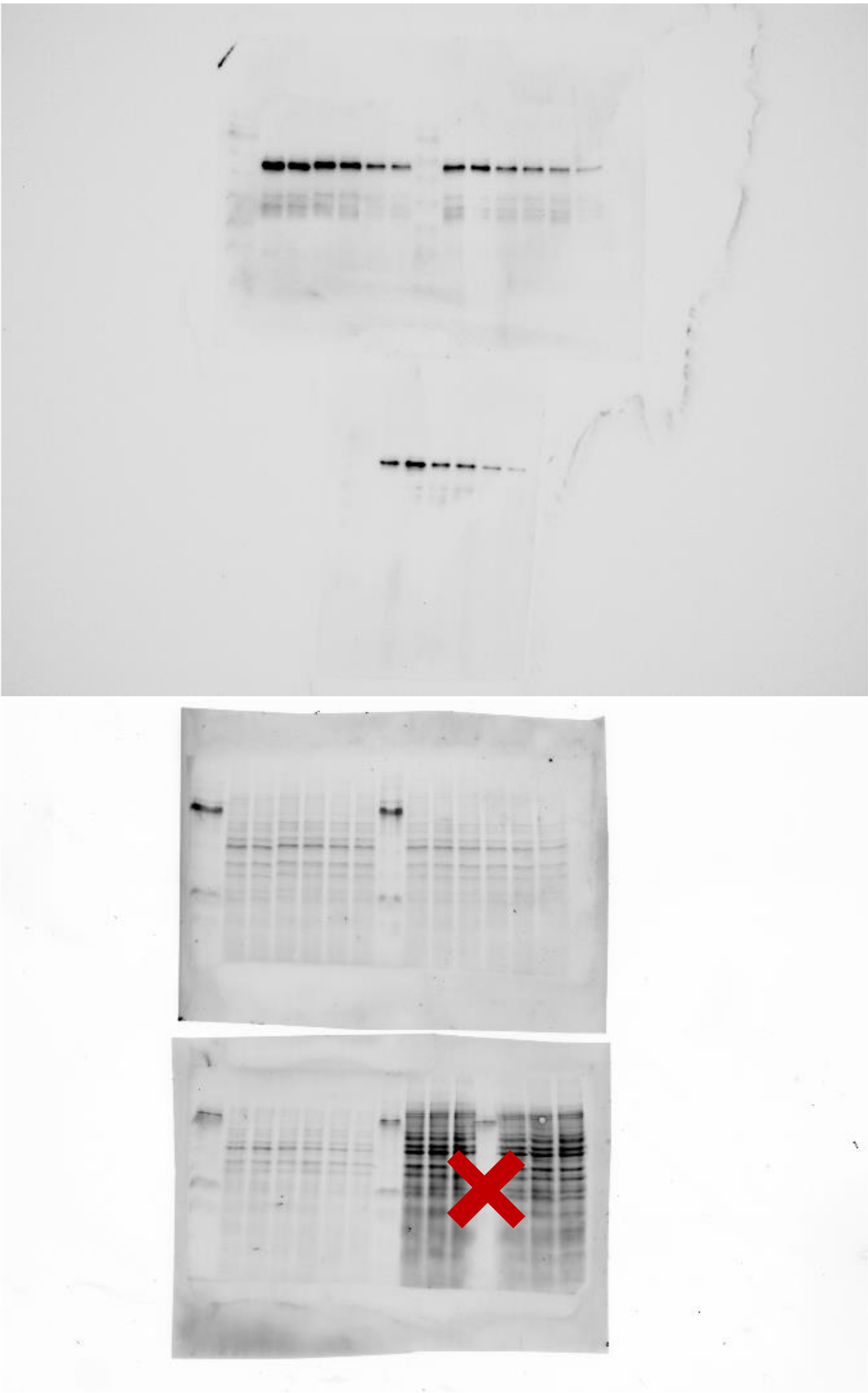

## Fig 5A

TIM44 TCL mito prots : SDHa

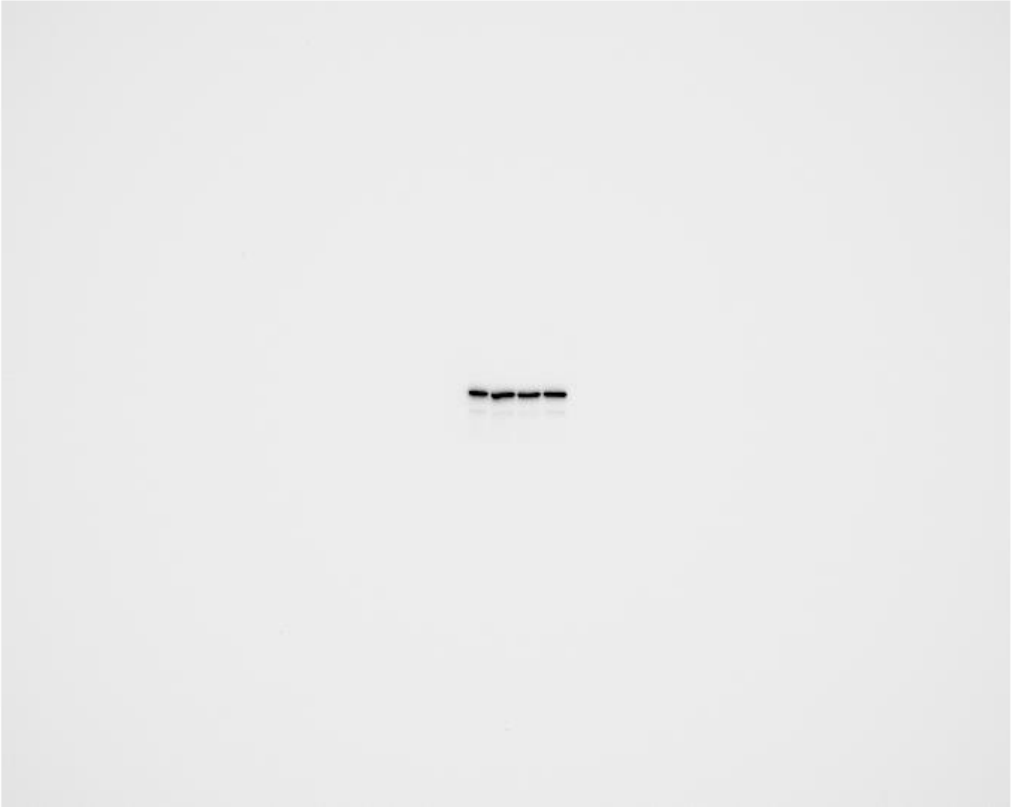

SOD2

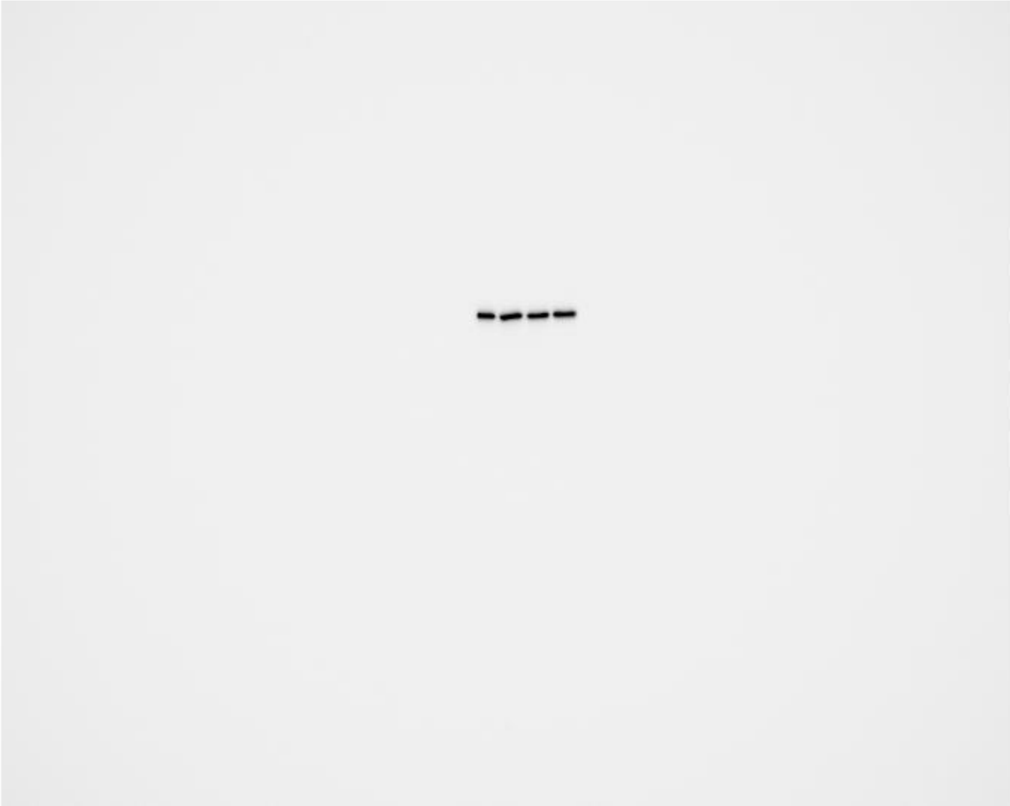

TOM20

UQCRC2

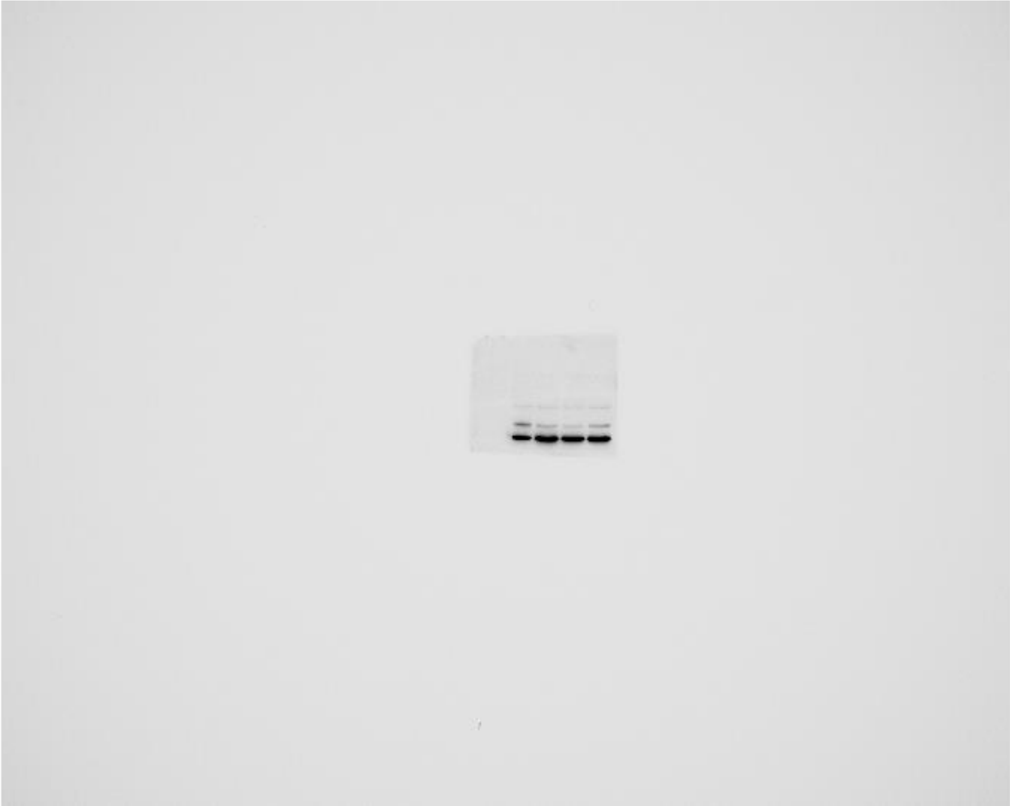

Cytc

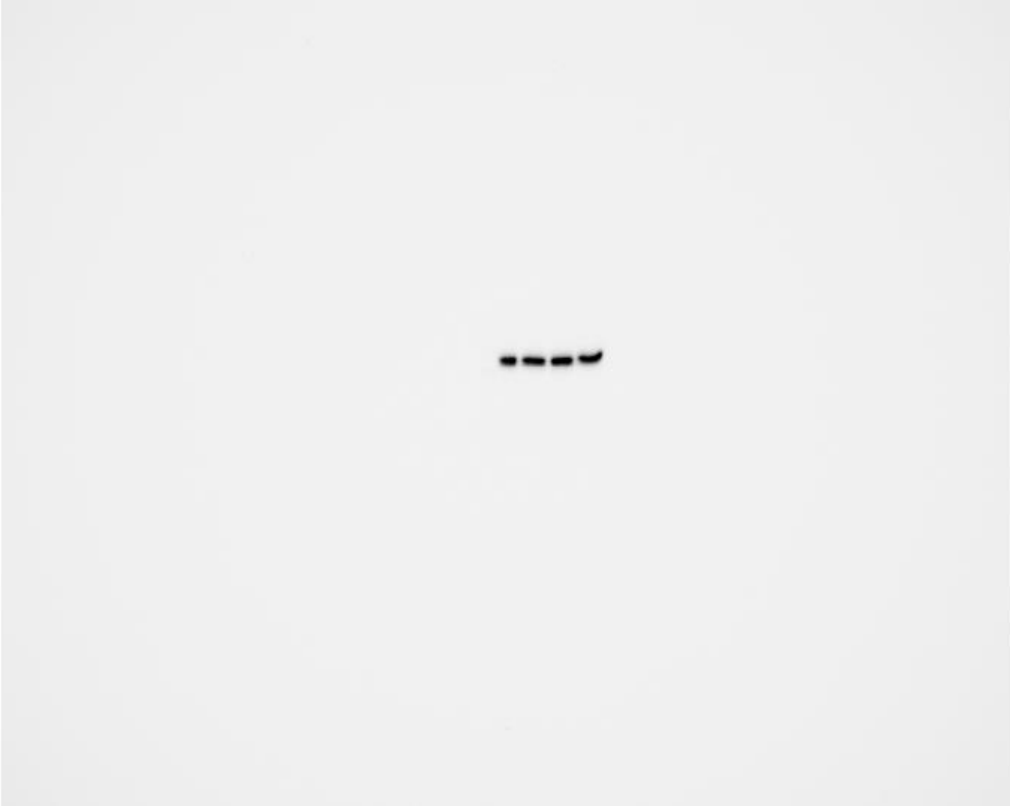

NDUFA9

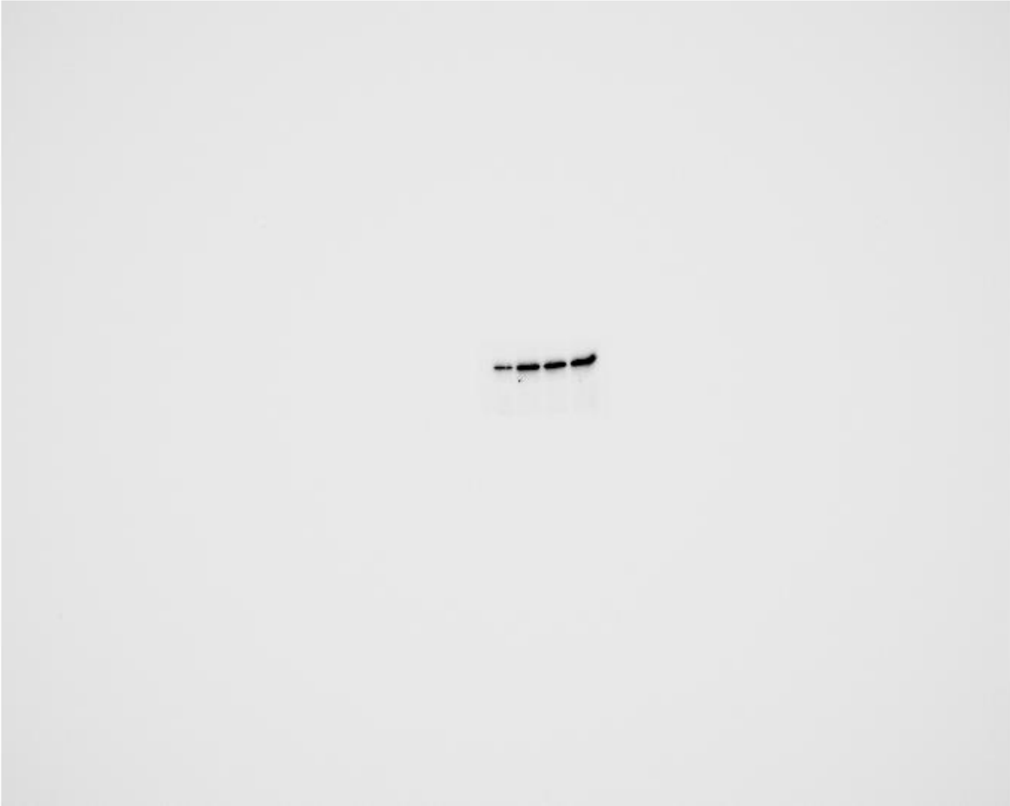

PAM16

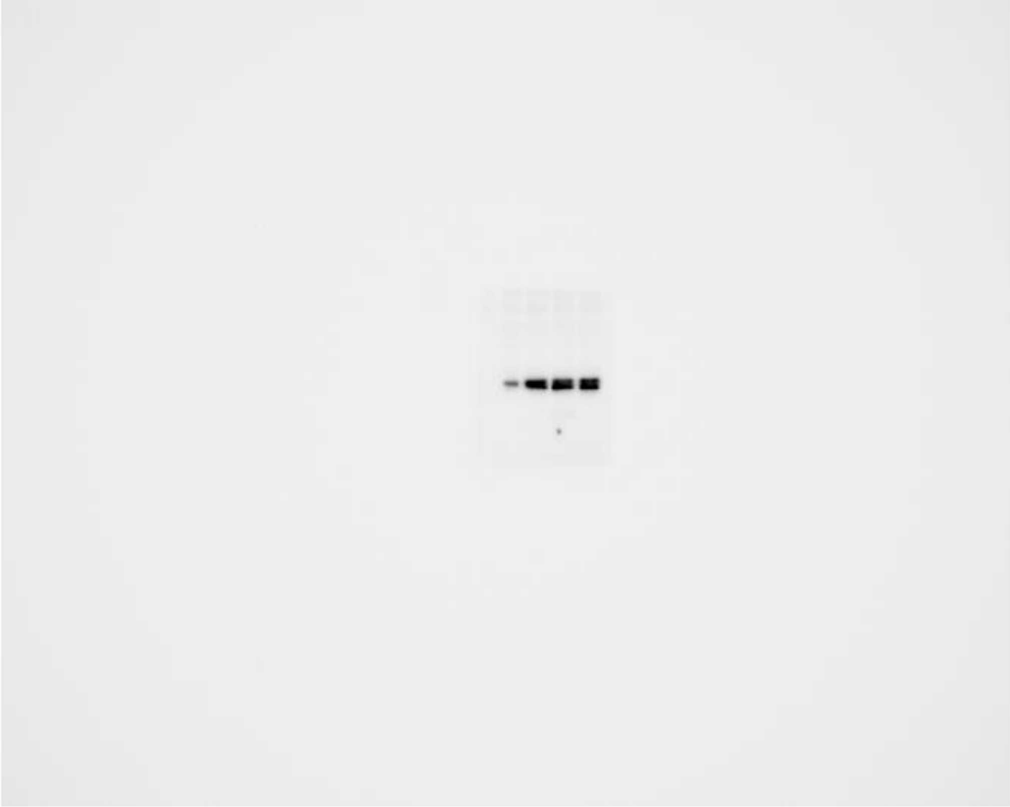

Hsp60

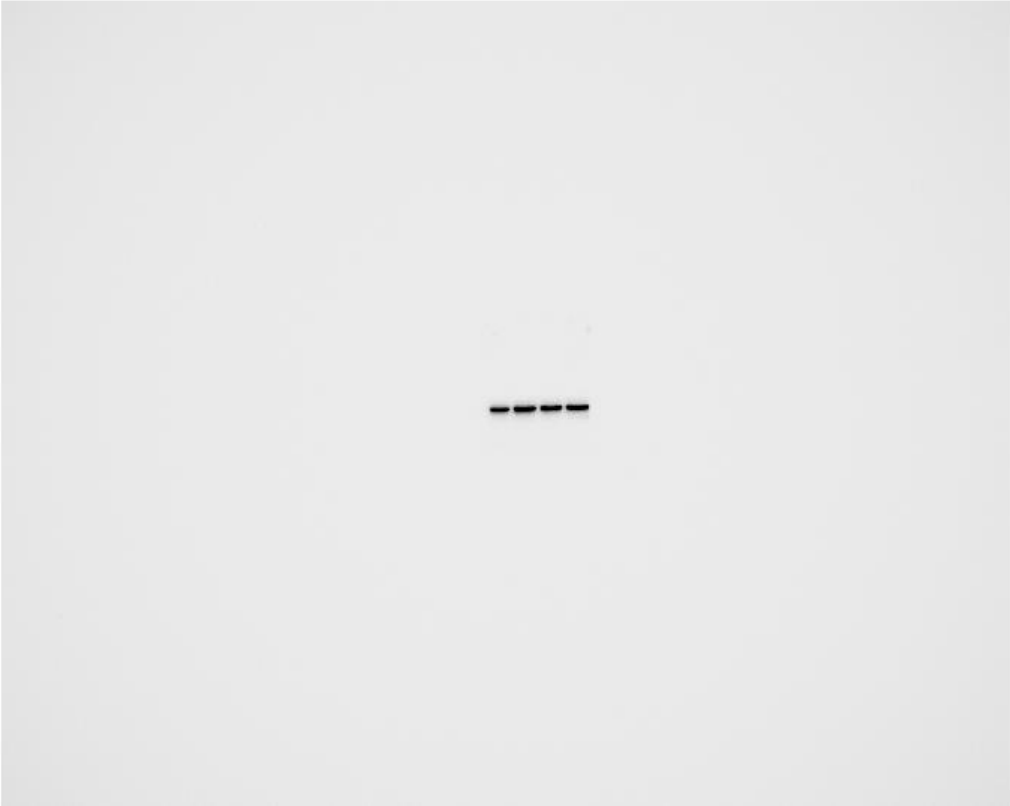

Hsp70

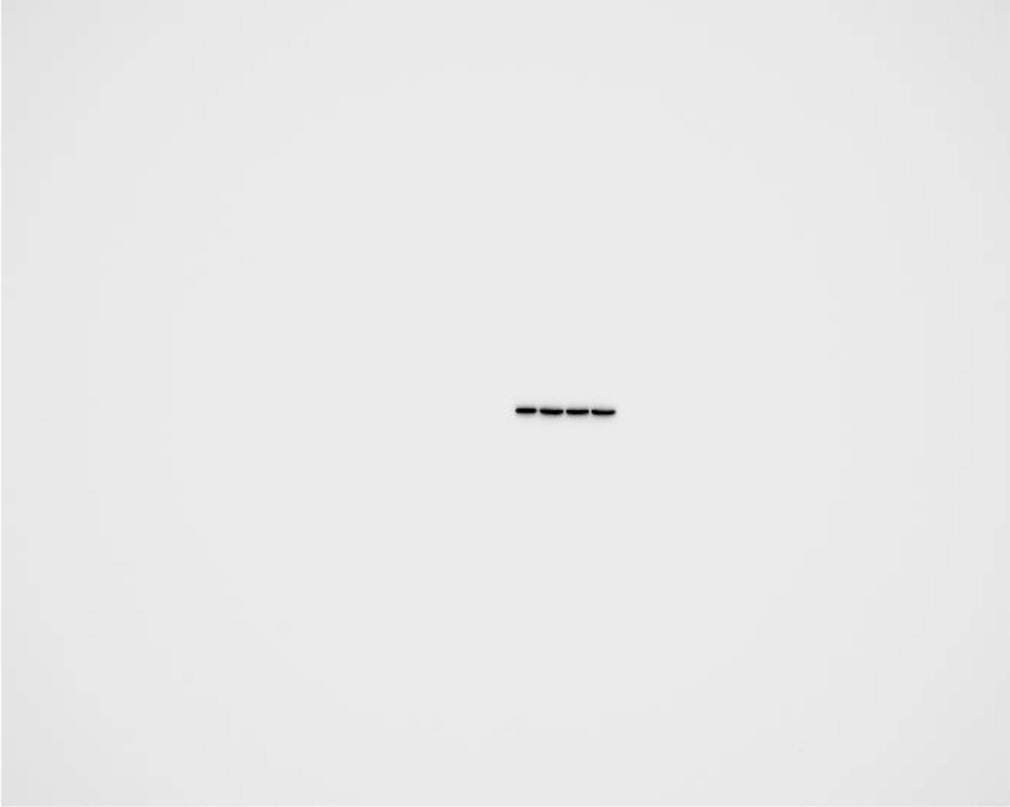

V5

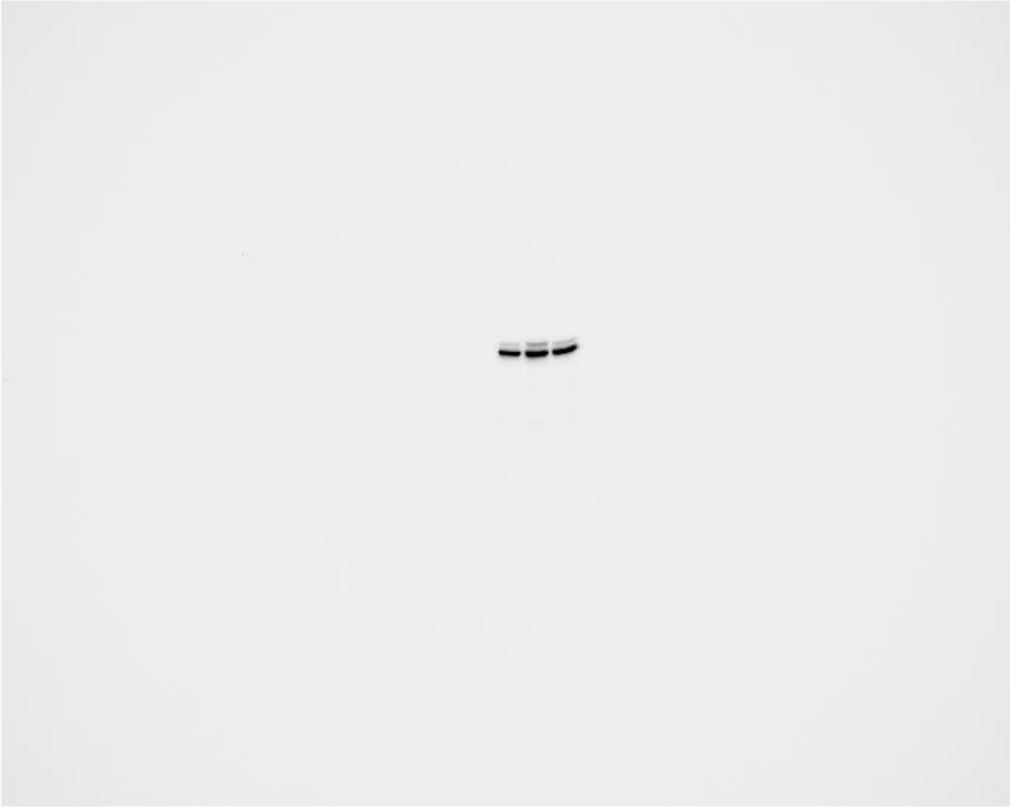

TPL

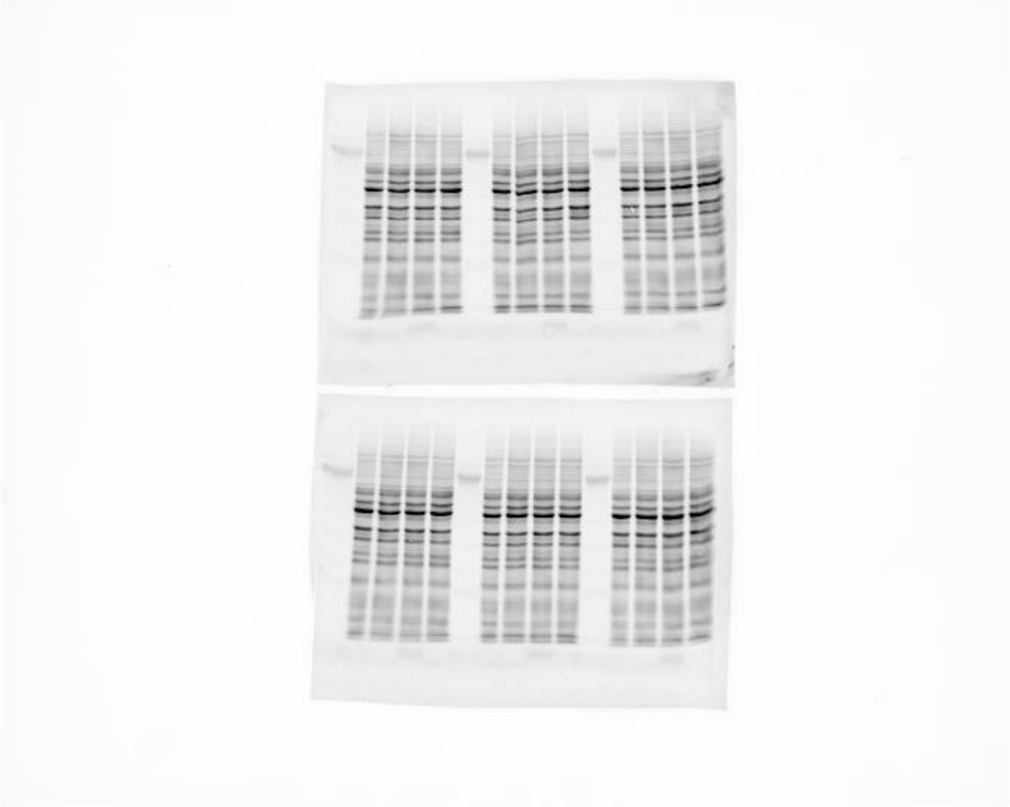

## Fig 6A

BN V5

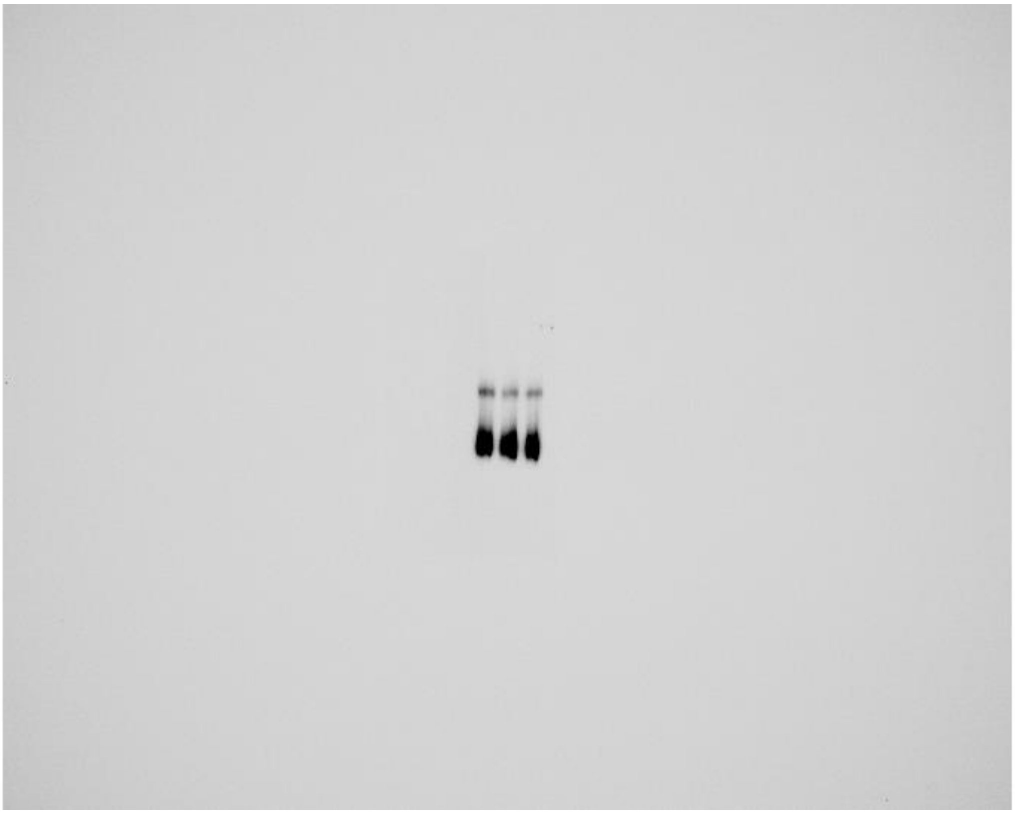

BN PKA Cat left

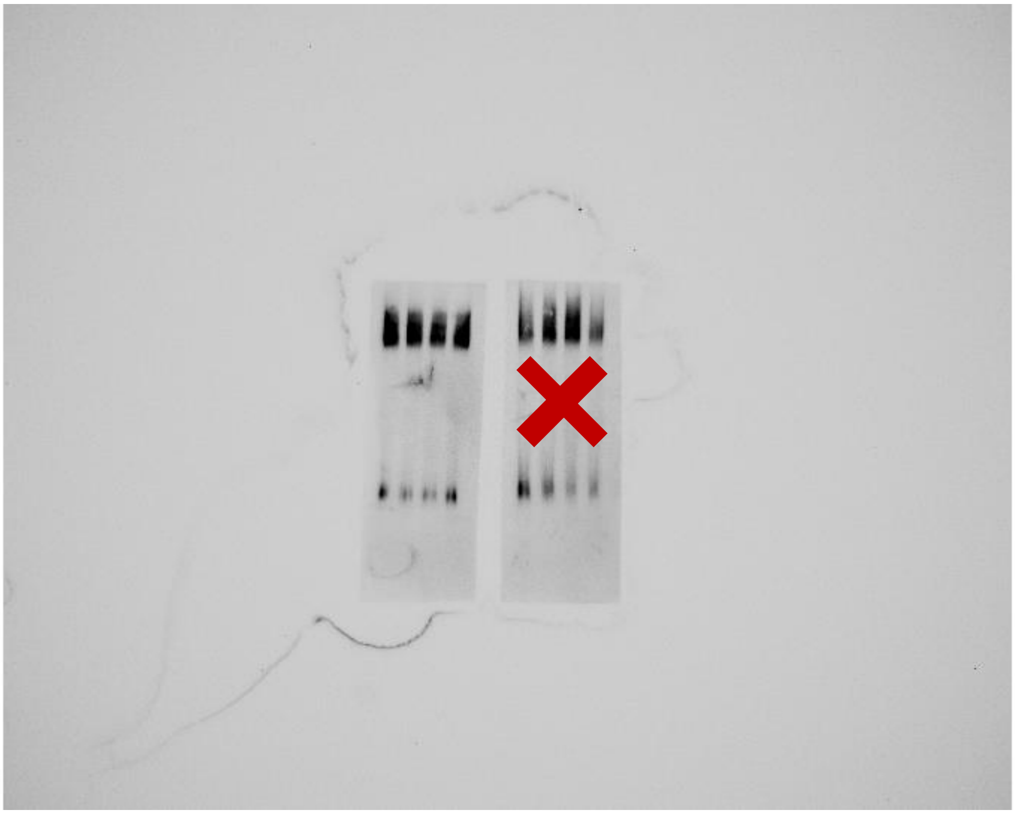

BN SDHa right

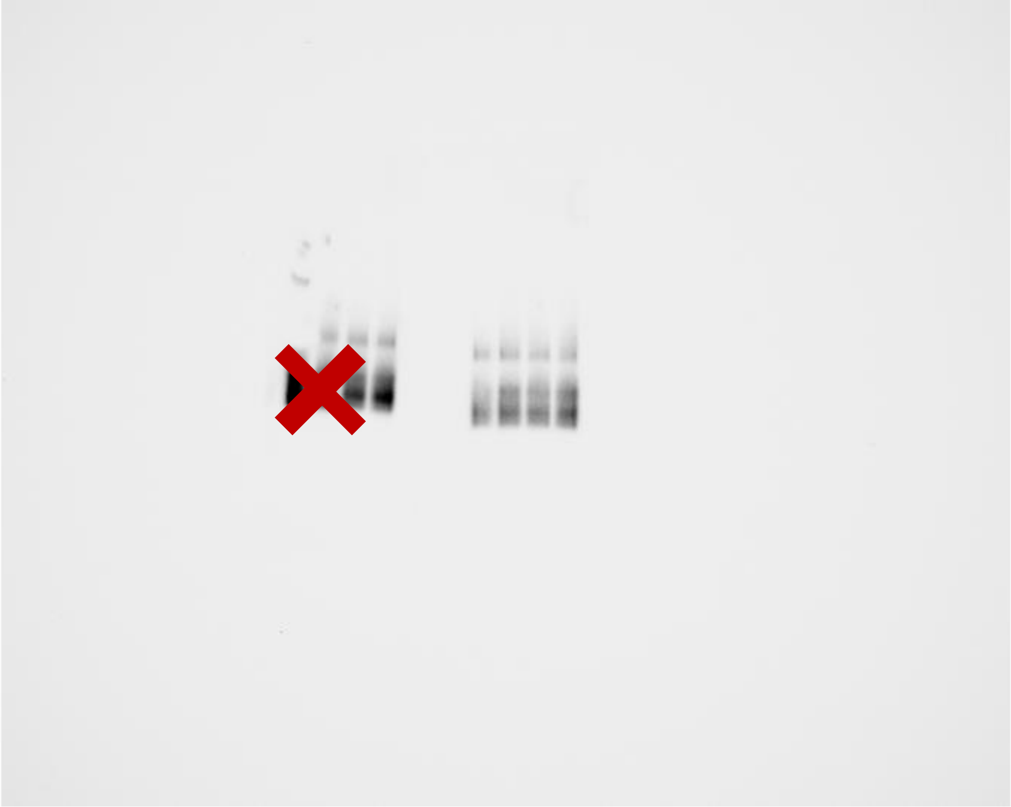

## Fig 6B

TCL TIM44 PKA Cat 3n rep on the right with Stain free right down

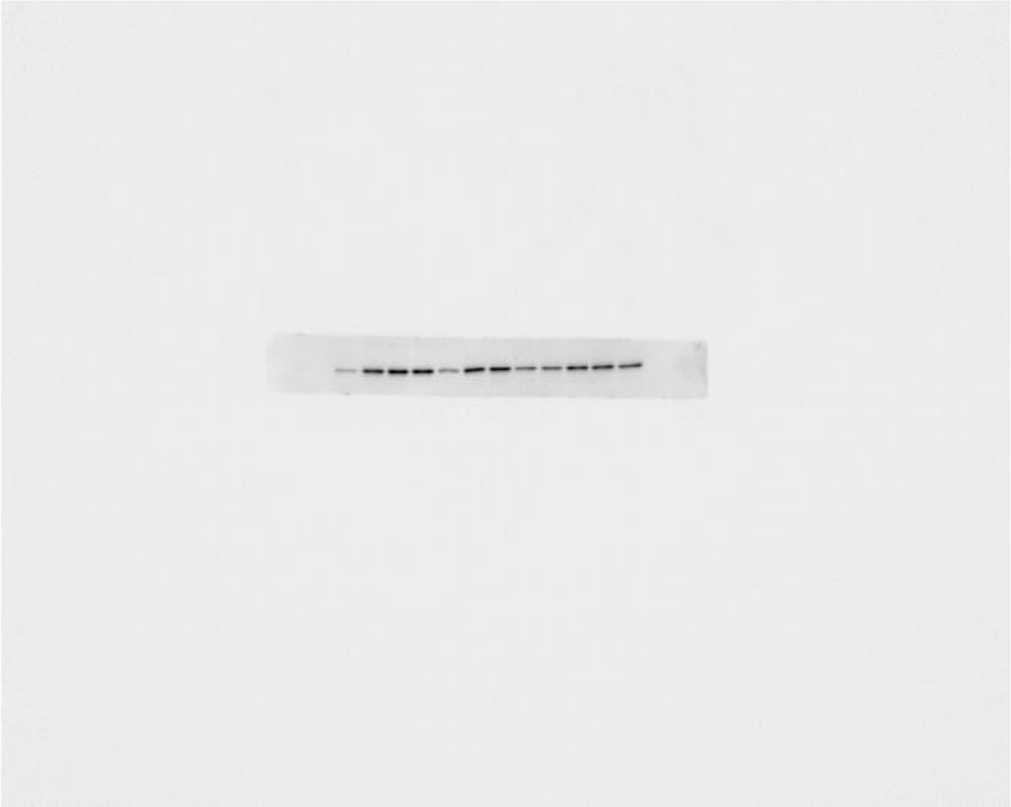

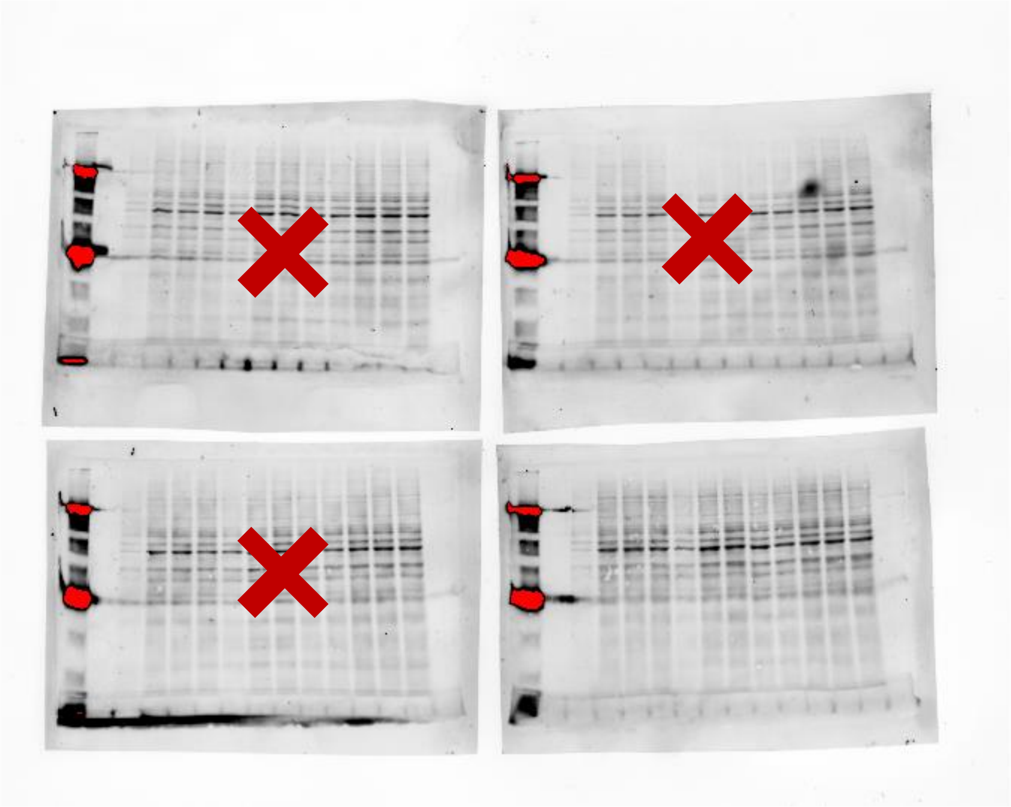

# Fig 6C

IP V5 anti V5

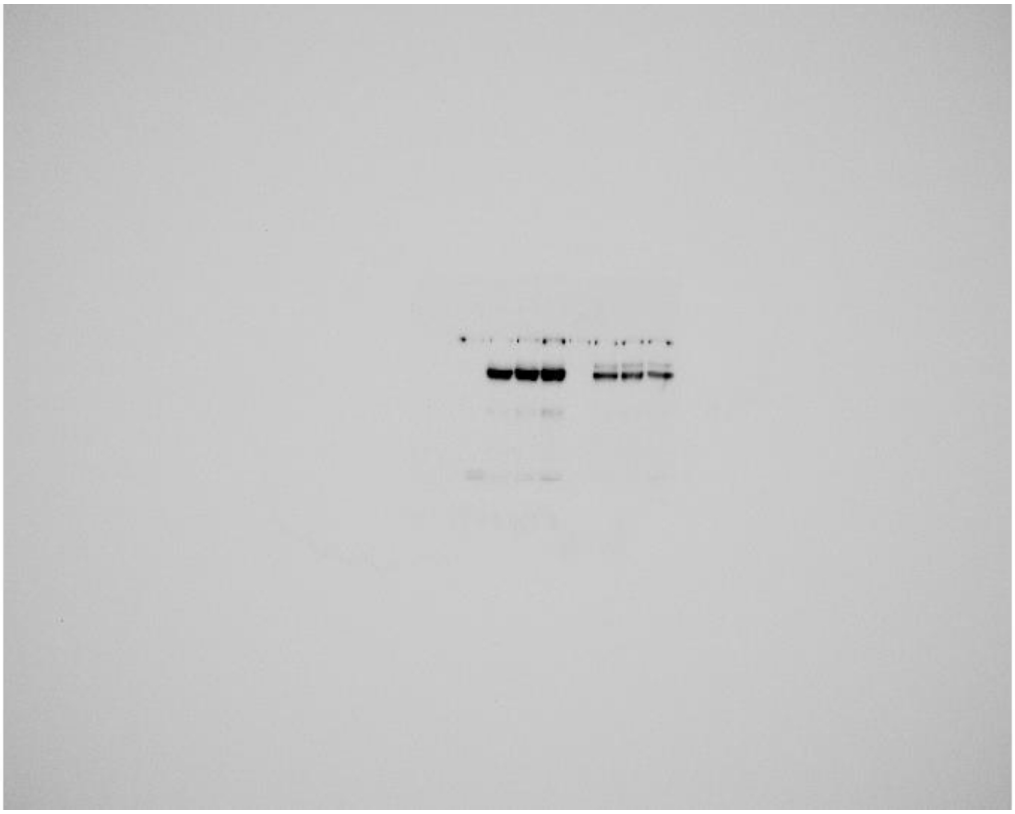

IP V5 anti Myc

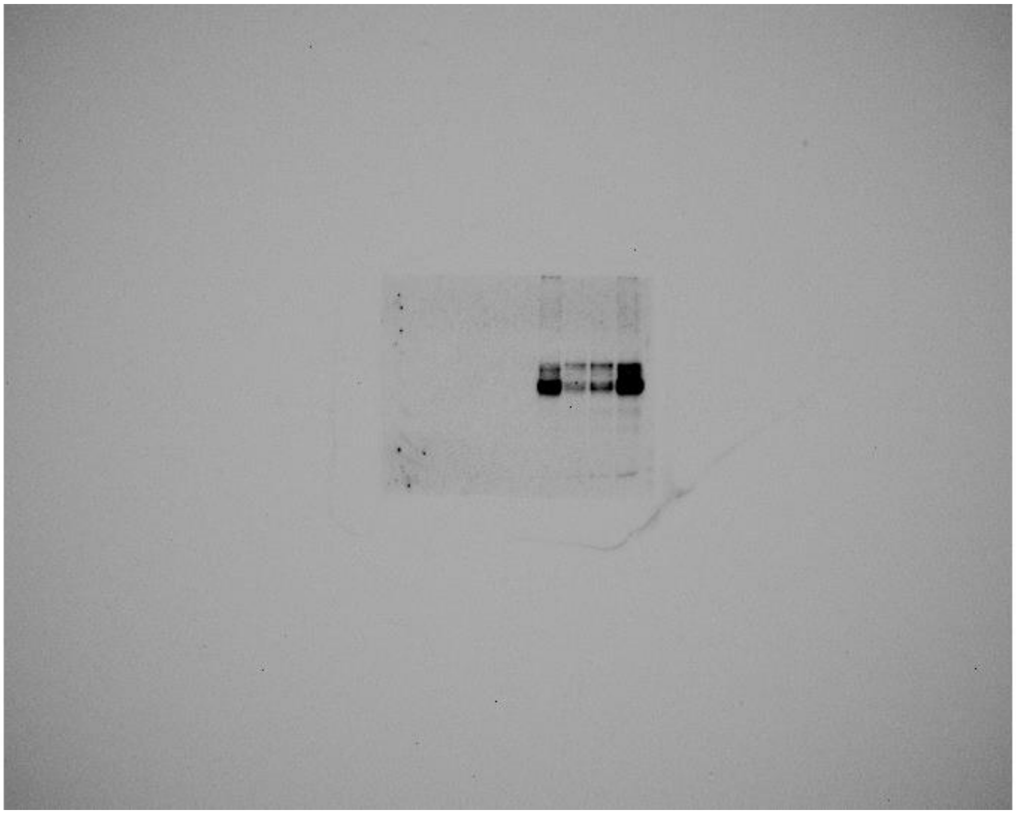

TCL V5

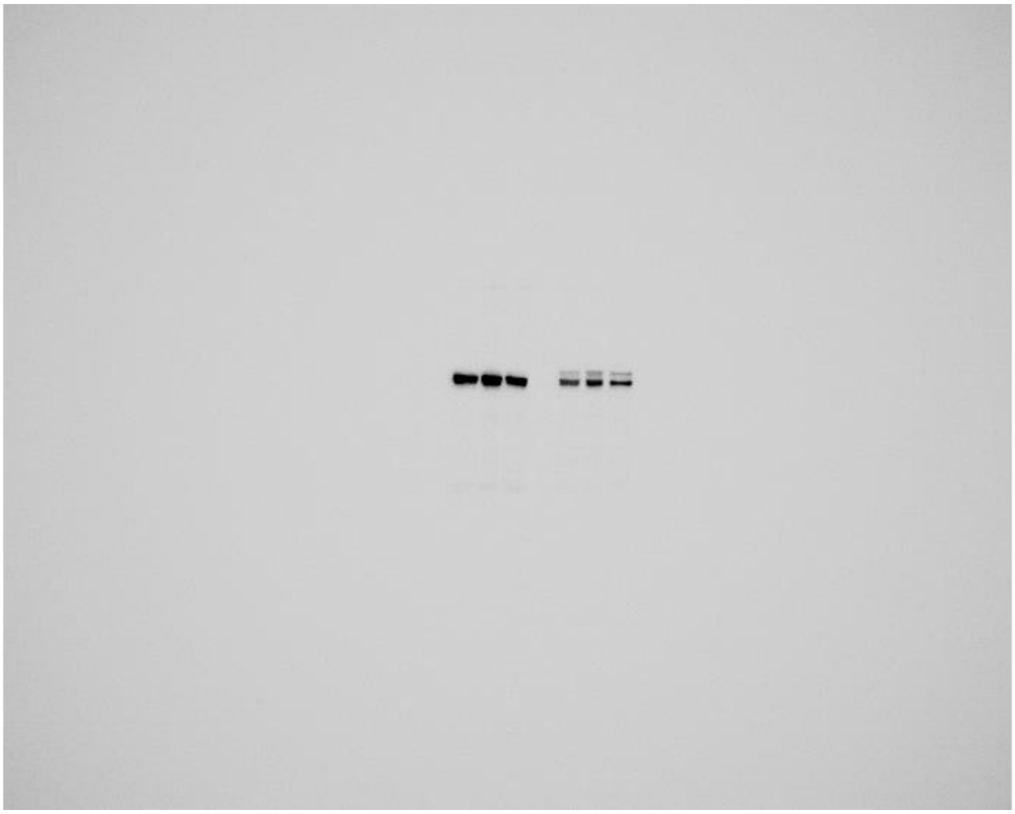

TCL Myc

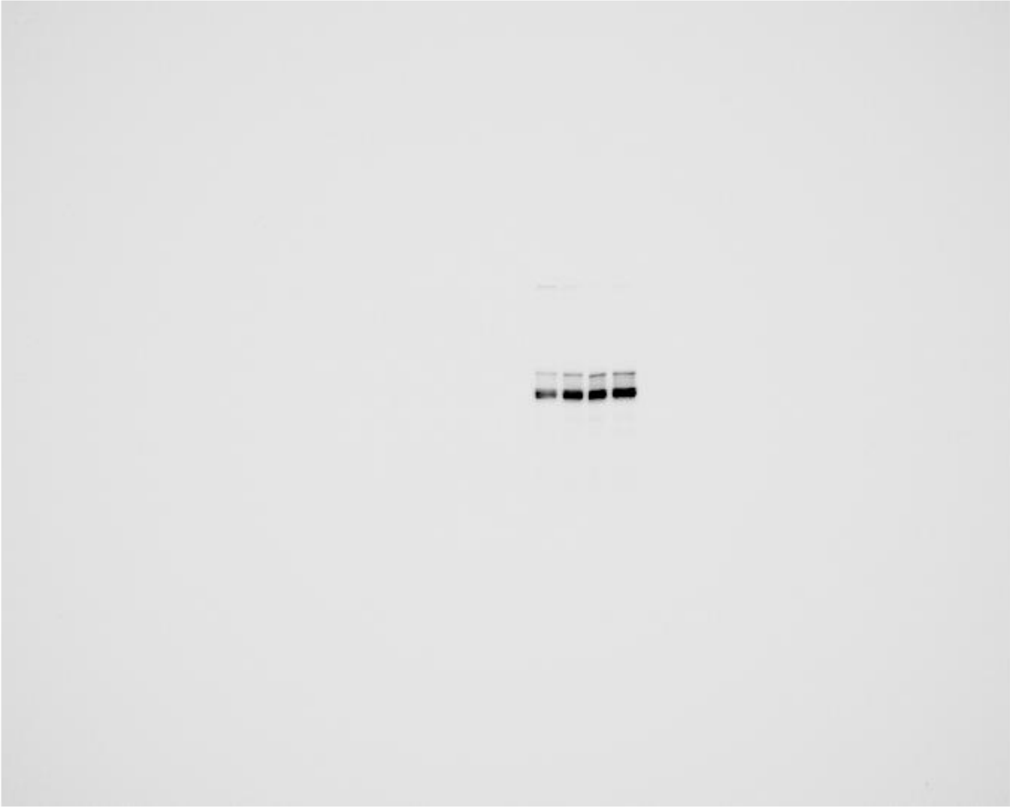

Stain free input left IP right

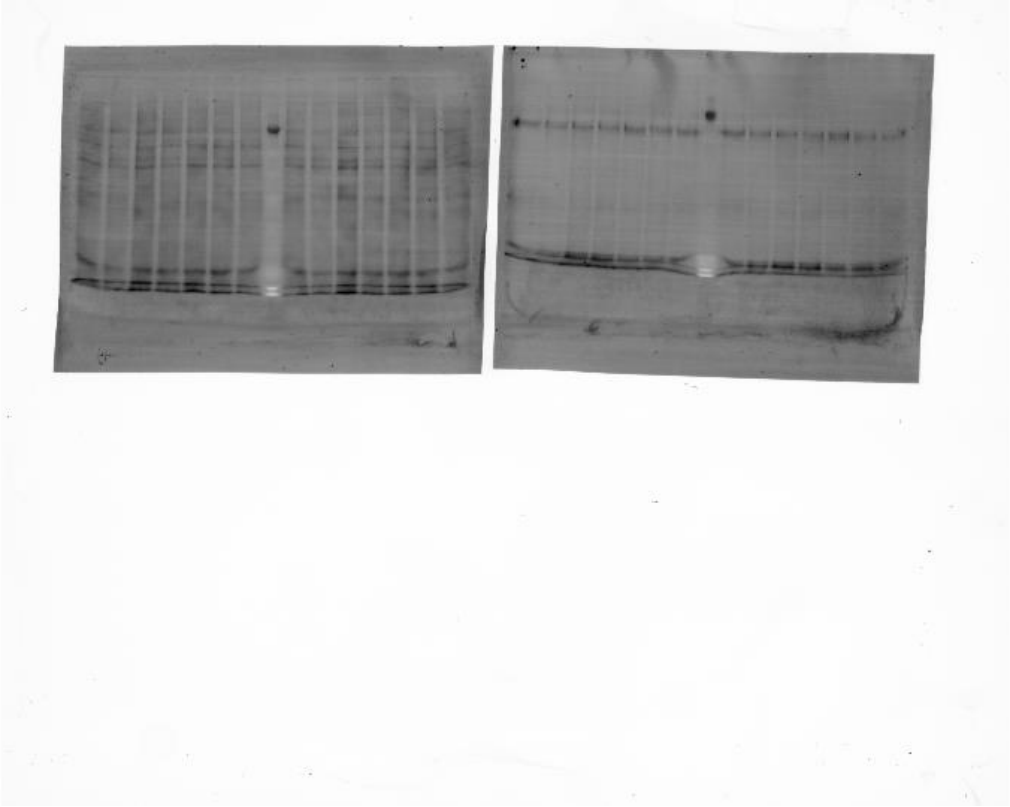

## Supplementary Fig. 1C

TCL TIM44 pPKA substrate  with Stain free  down right

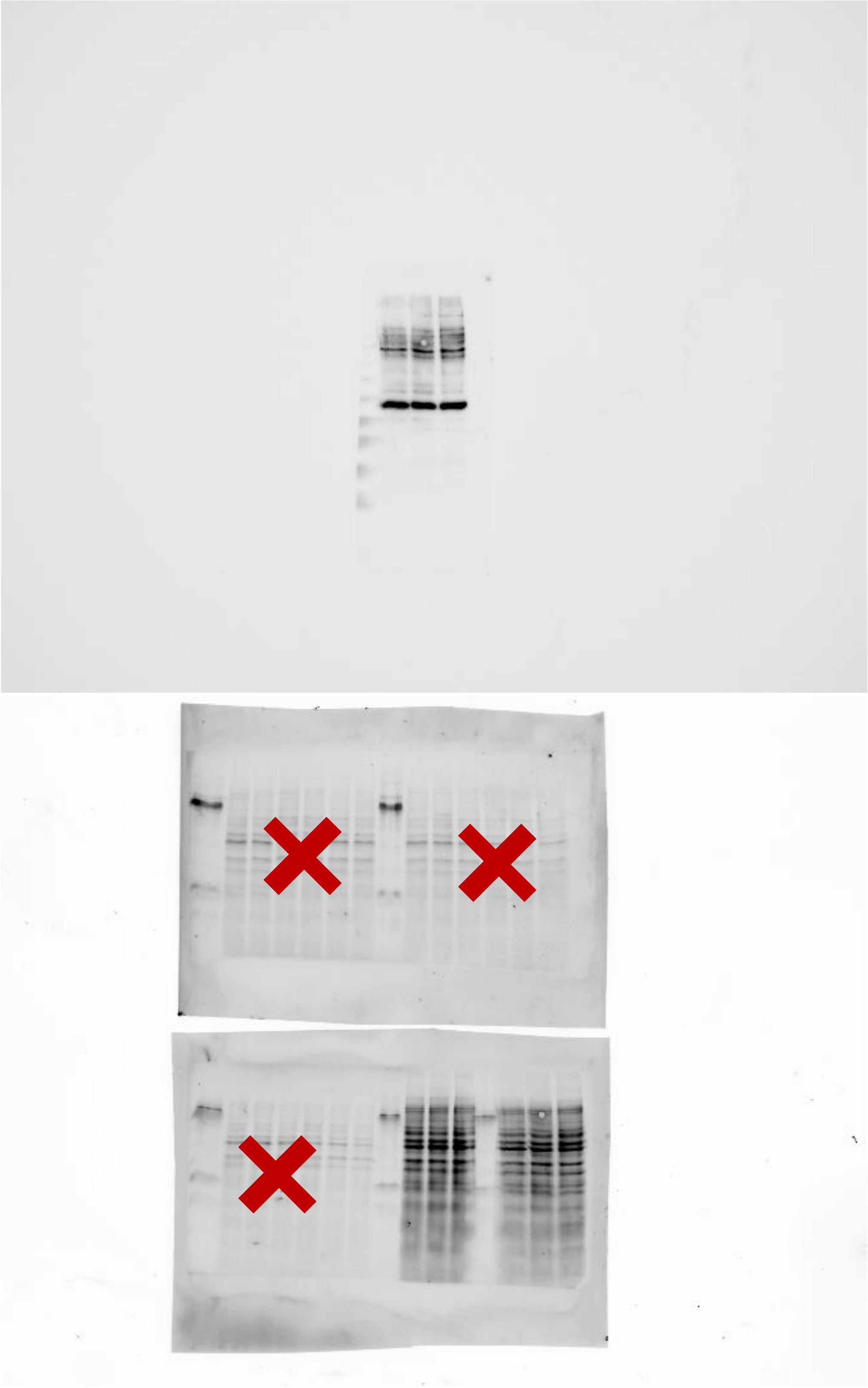

Supplement: Supplementary file 1 [file ijms-21-08283-s001.zip › Suppl material/Uncropped WB.pdf]
