# Supplementary figures and images for: Insight into the Interactome of Intramitochondrial PKA Using Biotinylation-Proximity Labeling
Source: Int J Mol Sci. 2020 Nov 5;21(21):8283. doi: 10.3390/ijms21218283 (PMC7663848; doi:10.3390/ijms21218283)

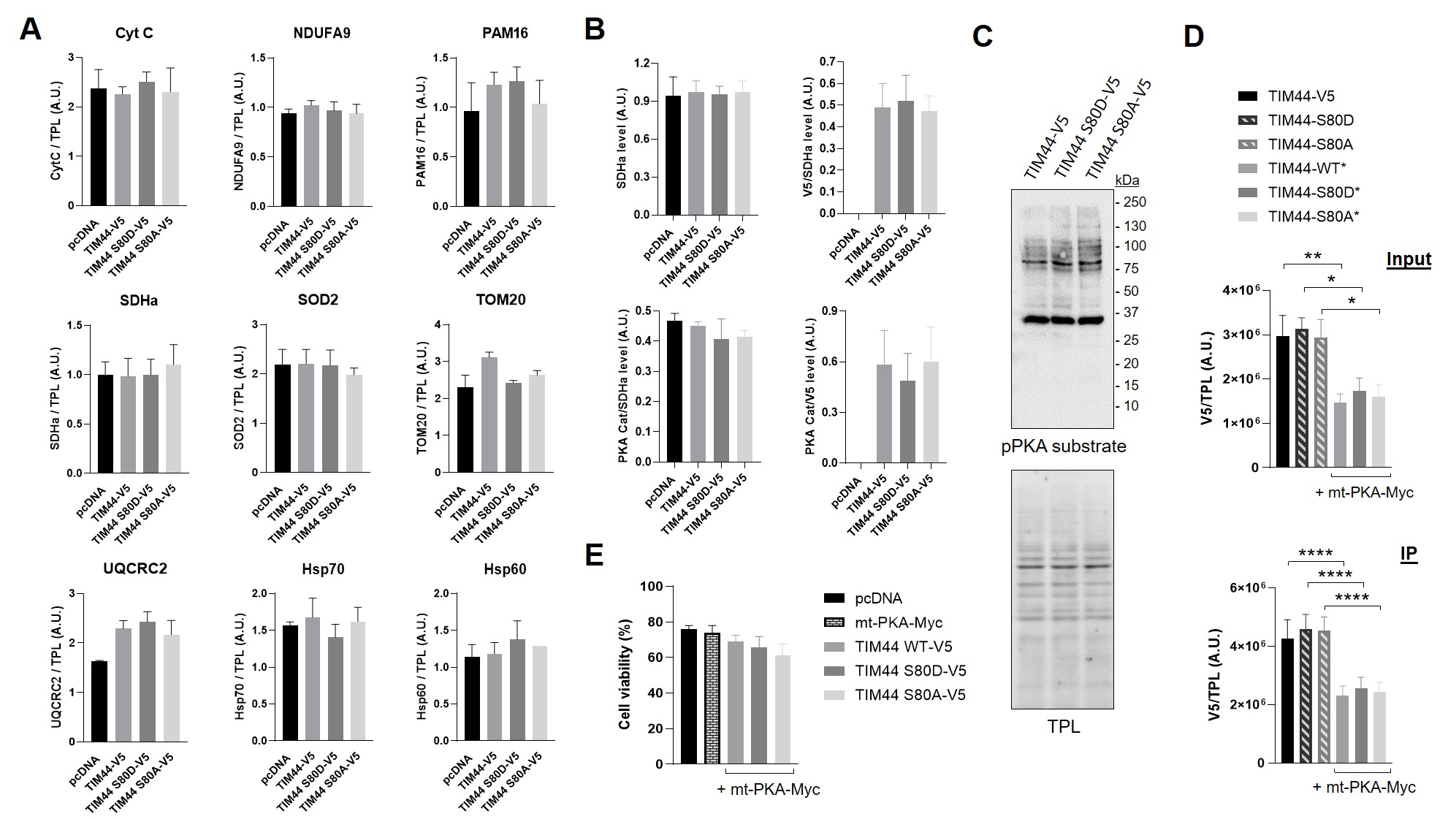

Supplement: Supplementary file 1 [file ijms-21-08283-s001.zip › Suppl material/Supplementary figure 1.jpg]
